# Bacterial and viral pathogen-associated molecular patterns induce divergent early transcriptomic landscapes in a bovine macrophage cell line

**DOI:** 10.1186/s12864-018-5411-5

**Published:** 2019-01-08

**Authors:** Felix N. Toka, Kiera Dunaway, Felicia Smaltz, Lidia Szulc-Dąbrowska, Jenny Drnevich, Matylda Barbara Mielcarska, Magdalena Bossowska-Nowicka, Matthias Schweizer

**Affiliations:** 1 Department of Biomedical Sciences, Center for Integrative Mammalian Research, Ross University School of Veterinary Medicine, 00-334 Basseterre, Saint Kitts and Nevis; 2 Department of Preclinical Sciences, Faculty of Veterinary Medicine, SGGW, Warsaw, Poland; 3 HPCBio and the Carver Biotechnology Center, University of Illinois, Champaign, IL USA; 4 Institute of Virology and Immunology, Federal Food Safety and Veterinary Office FSVO, Bern, Switzerland; 5 Department of Infectious Diseases and Pathobiology, Vetsuisse Faculty, University of Bern, Bern, Switzerland

**Keywords:** RNASeq, Bomac cells, Poly(I:C), CpG DNA, PAMPs, Bovine macrophage

## Abstract

**Background:**

Pathogens stimulate immune functions of macrophages. Macrophages are a key sentinel cell regulating the response to pathogenic ligands and orchestrating the direction of the immune response. Our study aimed at investigating the early transcriptomic changes of bovine macrophages (Bomacs) in response to stimulation with CpG DNA or polyI:C, representing bacterial and viral ligands respectively, and performed transcriptomics by RNA sequencing (RNASeq). KEGG, GO and IPA analytical tools were used to reconstruct pathways, networks and to map out molecular and cellular functions of differentially expressed genes (DE) in stimulated cells.

**Results:**

A one-way ANOVA analysis of RNASeq data revealed significant differences between the CpG DNA and polyI:C-stimulated Bomac. Of the 13,740 genes mapped to the bovine genome, 2245 had *p-*value ≤0.05, deemed as DE. At 6 h post stimulation of Bomac, poly(I:C) induced a very different transcriptomic profile from that induced by CpG DNA. Whereas, 347 genes were upregulated and 210 downregulated in response to CpG DNA, poly(I:C) upregulated 761 genes and downregulated 414 genes. The topmost DE genes in poly(I:C)-stimulated cells had thousand-fold changes with highly significant *p*-values, whereas in CpG DNA stimulated cells had 2–5-fold changes with less stringent *p*-values. The highest DE genes in both stimulations belonged to the TNF superfamily, *TNFSF18* (CpG) and *TNFSF10* (poly(I:C)) and in both cases the lowest downregulated gene was *CYP1A1*. CpG DNA highly induced canonical pathways that are unrelated to immune response in Bomac. CpG DNA influenced expression of genes involved in molecular and cellular functions in free radical scavenging. By contrast, poly(I:C) highly induced exclusively canonical pathways directly related to antiviral immune functions mediated by interferon signalling genes. The transcriptomic profile after poly(I:C)-stimulation was consistent with induction of TLR3 signalling.

**Conclusion:**

CpG DNA and poly(I:C) induce different early transcriptional landscapes in Bomac, but each is suited to a specific function of macrophages during interaction with pathogens. Poly(I:C) influenced antiviral response genes, whereas CpG DNA influenced genes important for phagocytic processes. Poly(I:C) was more potent in setting the inflammatory landscape desirable for an efficient immune response against virus infection.

**Electronic supplementary material:**

The online version of this article (10.1186/s12864-018-5411-5) contains supplementary material, which is available to authorized users.

## Background

Gene expression profiles generated from a wide variety of cell types treated with different pathogens or substances that mimic pathogens can yield valuable insights into the mechanisms of host-pathogen interaction. Moreover, gene expression profiles permit identification of specific responses to microbial stimuli that can lead to rationale design of therapeutic approaches or next-generation vaccines. With the completion of the bovine genome [1], it is possible to analyze transcriptional profiles or interactomes to understand the host-pathogen relationships that lead to cellular responses. Therefore, highly sophisticated techniques such as RNASeq have become useful to study whole transcriptomic landscapes of cells during infection or treatment with therapeutic agents. There is now abundant evidence that the innate immune response immensely influences the development of an infection into disease. Among other cells at the forefront of innate immunity, macrophages (MΦ) play a major role in containing primary infections. For instance, infection of mice with herpes simplex 1 virus followed by induction of M2 macrophages through CSF-1 DNA was associated with reduced virus replication in the eye, reduced latency and reduced levels of CD4, CD8, IFN-γ and PD-1 transcripts in the trigeminal ganglion [2]. Some reports show that MΦ alter their gene expression profile upon infection, e.g., *Mycobacterium bovis* infection is associated with the repression of host gene expression in MΦ [3, 4]. Therefore, reaction of MΦ to various pathogens is variable and is not yet completely understood. Lewandowska-Sabat et al. [5] have reported the early phase transcriptional program of bovine monocyte-derived MΦ infected with *Staphylococcus aureus* and show that *S. aureus* induces both, alternative and classical MΦ activation pathways. They concluded that activation of MΦ through the alternative pathway possibly contributes to intracellular persistence of *S. aureus* during mastitis in dairy cattle. Infection of an epithelial cell-MΦ co-culture with *Mycobacterium avium* subspecies *paratuberculosis* (MAP) revealed a number of metabolic, DNA repair and virulence genes that are worthy to investigate for new drug targets [6]. In particular, this study revealed a novel iron assimilation system for carboxymycobactin. Another RNASeq study of MAP infection [7] of monocyte-derived MΦ showed expression of genes that account for protective host immunity and those that might support MAP survival and proliferation in MΦ.

Antigen presenting cells (APCs), such as MΦ, express pattern recognition receptors (PRRs) such as Toll-like receptors (TLRs), which are used for detecting pathogen-associated molecular patterns (PAMPs). PRR signal through intermediate molecular adaptors to activate transcription factors that drive gene transcription and expression of pro-inflammatory cytokines responsible for antimicrobial activity. CpG DNA and synthetic dsRNA (poly(I:C)) are classically used as model ligands to represent specific pathogens such as bacteria and dsRNA viruses, and are potent adjuvants of immune response in many animal species. Therefore, it is imperative that the influence of adjuvants on the responding cells be thoroughly understood. During stimulation with dsRNA, the adaptor TRIF (TIR-domain-containing adapter-inducing interferon-β) is recruited to the Toll/Il-1 Receptor (TIR) domain of activated, dimeric TLR3, which leads to stimulation of the transcription factors IRF3, NF-κB and AP-1 [8, 9]. By contrast, the chaperon protein UNC93B mediates transport of TLR9 to the endosome where the binding with DNA molecules induces the formation of the signalling complex that includes MyD88, TRAF6 and IRAK4. The resulting response activates the NF-κB pathway leading to the production of pro-inflammatory cytokines. In addition, activation of IRF7 upon TLR9 activation rather leads to the production of type-I and -III interferon (IFN) which depend on numerous co-activators such as IRAK1, TRAF3, OPN, IKKα and PI3Kδ [10].

Bomac cells are often used but the responsiveness to CpG DNA and poly(I:C) has not been previously assessed using high throughput genomics techniques. To shed more light on the initial transcriptional response of an innate immune cell upon encounter with different PAMPs, we studied the influence of CpG DNA and poly(I:C) on a bovine MΦ cell line. Thus, the purpose of this study was to examine differential expression of genes in bovine macrophages under the influence TLR3 and TLR9 agonists in order to better understand the early phase of transcriptional changes induced by CpG DNA or poly(I:C) in the Bomac macrophage cell line. Our results show that poly(I:C) induces a transcriptional profile rather resembling an antiviral immune response, whereas CpG DNA induces a varied transcriptional profile with a highly marked function of free radical scavenging.

## Methods

### Cells

The Bomac cell line is a transformed bovine peritoneal macrophage cell line described by Stabel and Stabel [11]. The original Bomac cell line present in the laboratory of one of the authors [12] was found to be contaminated with bovine viral diarrhea virus (BVDV), probably present already in the original cell type. The cell line was subsequently cured of BVDV (Marti, S. and Schweizer, M., manuscript in preparation; [13], and maintained in IMDM supplemented with Glutamax, Hepes, 10% fetal calf serum, non-essential amino acids, MEM vitamins, penicillin and streptomycin, and 2-mercaptoethanol. The cured cell line used in these studies was obtained from Dr. Matthias Schweizer. Cells were cultured at 37 °C in 5% CO_2_ in the atmosphere and subcultured every two days. Additionally, the cells were tested for Mycoplasma contamination using the PlasmoTest™ Mycoplasma Detection Kit from Invivogen, and found to be negative (Additional file 1). Further, a conventional RT-PCR was performed to detect BVDV in the cured cells. We did not detect and BDVD sequences (Additional file 1).

#### Flow cytometry

Because the Bomac cells have been reported not to be a homogeneous population, and have lost certain functions, we sought to determine the phenotype of the cells that we were working with, by flow cytometry. Bomac cells were stained with anti-CD14 (cat. nr MCA2678GA), CD16 (cat. nr MCA5665GA), CD11b (cat. nr MCA1425F), CD172a (cat. nr MCA6079), CD44 (cat. nr MCA2433F), MHC II (cat. nr MCA2445PE) from BioRad, and CD40 (cat. nr ABIN2480301), CD68 (cat. nr ABIN2472322), CD71 (cat. nr ABIN2560526), CCR2 (ABIN2787667) from Antibodies Online. Another set of cells were stimulated with p(I:C) as described in the Cell stimulation section, and then similarly stained for flow cytometry. At least 100,000 events were acquired on a BD FACS Calibur flow cytometer, and later analyzed in FlowJo. A total of six determinations were performed and means compared using the descriptive statistical analysis tool in Microsoft Excel.

### Cell stimulation

Cells were first cultured in 6 well plates at a density of 10^4^ cells per well for 24 h at the time they reached confluence. The maintenance medium was then removed and replaced with medium containing CpG DNA or poly(I:C) at 10 μg/ml or 50 μg/ml, respectively. A separate culture plate was set with unstimulated cells to serve as control. We used the class B CpG oligonucleotide (ODN 2007) reactive with bovine TLR9, whereas the poly(I:C) was a low molecular weight synthetic analog of dsRNA specific for TLR3. Both reagents were obtained from InvivoGen (San Diego, USA). In order to analyze the early phase of transcriptomic changes, cells were incubated in the presence of PAMPs for only 6 h prior to RNA isolation. This time point was chosen based on ELISA assays measuring the earliest appearance of IL-1β, IL-6 and IFNβ protein in the supernatants following stimulation with CpG DNA or poly(I:C). For that purpose, Bomac cells, plated in 6-well plates, were stimulated with the individual PAMPs and incubated for 2, 4, 6, 8 and 12 h prior to collecting the cell culture supernatants and quantification of the cytokines by ELISA using reagents purchased from BioSource according to the manufacturers’ instructions. The first cytokines to be detected were IL-6 and IFNβ at 8 and 12 h post stimulation, whereas IL-1β protein was not detectable at time points earlier than 18 h (data not shown). Therefore, we selected 6 h as the earliest time point to track early transcriptomic changes in Bomac cells stimulated with PAMPs.

### RNA isolation

After the 6 h incubation period, the maintenance medium was removed from the wells containing stimulated cells followed by two washes with cold phosphate-buffered saline. Cells were lyzed with Qiazol directly in the wells and lysates were transferred to DNase/RNase free microfuge tubes for further processing. RNA isolation was carried out as described [14] and according to the manufacturers’ instructions. The Qiagen miRNeasy Mini kit was used for RNA isolation. RNA concentration was measured using the Implen NanoPhotometer (Implen GmbH, Munich, Germany). RNA integrity was monitored by agarose RNA electrophoresis (Additional file 2). RNA samples were dried in GenTegra-RNA tubes (GenTegra, USA) using a VentriVap DNA concentrator (Labconco, USA) for 55 min. at 25–27 °C and sent for sequencing. Each stimulation was repeated in three separate experiments and, thus, 9 RNA samples (control, CpG DNA-stimulated, poly(I:C)-stimulated cells) were submitted for RNASeq.

### mRNA - sequencing and analysis

The samples were sequenced at the Roy J. Carver Biotechnology Center at the University of Illinois, USA. Ribosomal RNA was removed with the Ribozero Human/mouse/rat kit (Illumina, USA). Strand-specific single-end libraries were prepared using the TruSeq Stranded mRNA-Seq Sample Prep kit (Illumina, USA). The libraries were quantitated by qPCR, pooled in equimolar amounts and sequenced on one lane of a HiSeq 4000 (Illumina; sequencing kit v 1), generating over 373 million 100 bp single-end reads. Fastq files were generated and demultiplexed per sample with the bcl2fastq v2.17.1.14 Conversion Software (Illumina), which also trims Illumina adapters from the reads and removes any resulting sequences shorter than 35 bp. All bases across the reads showed quality scores greater than Q30 (FASTQC v 0.11.2) and, thus, quality trimming was not performed. The *Bos taurus* UMD 3.1.1 reference genome was downloaded from NCBI along with Annotation release 105 gene models containing 35,315 Entrez Gene IDs. The gene models were converted from gff3 to gtf format using the rtracklayer package [15] (v 1.36.3) in R [16] (v 3.4.0) while extracting the Entrez Gene IDs to an attribute named “gene_id”. Reads were aligned to the genome using STAR (version 2.5.2b) [17] with parameters --sjdbGTFfeatureExon exon, −-sjdbGTFtagExonParentGene gene_id and --sjdbOverhang 99. Read counts per gene were generated using featureCounts [18] from the subread package [19] (v 1.5.0). The datasets generated for this study can be found in the NCBI, GEO accession number GSE106843.

The read counts were put into R [16] (v 3.4.0) for data pre-processing and statistical analysis using packages from Bioconductor [20] as indicated below. The samples had 23–36 million reads assigned to genes (Table 1), thus any gene without 0.5 Count Per Million (CPM) reads in at least 3 samples were filtered out. 13,740 genes passed this filter and were analyzed using edgeR [21] (v 3.18.1) in the quasi-likelihood pipeline [22, 23] that also accounted for the total library size for each sample and an extra TMM normalization factor [24] for any biases due to changes in total RNA composition of the samples. A one-way ANOVA test was employed from the model, along with pairwise comparisons between the two stimulation groups. Adjustment for multiple testing was done using the False Discovery Rate (FDR) method [25].

**Table 1:** Statistics of categorization and abundance of sequence reads generated from 9 cDNA libraries used for differential gene expression analysis

Sample Name	Assigned reads (millions)	Aligned reads (millions)	Unassigned Ambiguity	Unassigned Multi-mapping (millions)	Unassigned No-feature (millions)
1_Control_Expt1	30.8 (60.3%)	37.0 (84.7%)	31,750 (0.1%)	14.1 (27.6%)	6.1 (12%)
2_Control_Expt2	36.3 (58.4%)	43.2 (83.4%)	37,638 (0.1%)	18.9 (30.5%)	6.8 (11%)
3_Control_Expt3	33.2 (57.8%)	39.2 (82.6%)	34,731 (0.1%)	18.6 (31.7%)	6.0 (10.5%)
4_pI_C_Expt1	24.5 (57.4%)	29.1 (83.0%)	42,708 (0.1%)	13.5 (31.8%)	4.6 (10.7%)
5_pI_C_Expt2	29.2 (62.3%)	34.4 (85.8%)	52,978 (0.1%)	12.5 (26.7%)	5.1 (10.9%)
6_pI_C_Expt3	23.0 (59.3%)	27.4 (83.9%)	41,625 (0.1%)	11.5 (29.5%)	4.3 (11.1%)
7_CpG_Expt1	27.4 (58.0%)	32.9 (83.9%)	27,411 (0.1%)	14.3 (30.3%)	5.5 (11.6%)
8_CpG_Expt2	24.8 (55.9%)	29.8 (81.7%)	25,395 (0.1%)	14.5 (32.7%)	5.0 (11.3%)
9_CpG_Expt3	27.3 (37.8%)	32.8 (69.6%)	27,623 (0.0%)	39.4 (54.6%)	5.4 (7.5%)

Gene Ontology terms for the *B. taurus* genes were analyzed from Bioconductor’s org.Bt.eg.db (v 3.4.1) database while the Kyoto Encyclopedia of Genes and Genomes (KEGG) pathway mappings were performed directly from KEGG (http://www.genome.jp/kegg/) on July 12, 2017 using Bioconductor’s KEGGREST package (v 1.16.0). Overrepresentation testing for GO and KEGG terms was done using the goana() and kegga() functions from edgeR [21], which correct for any trends due to expression level [26]. Separate tests were done for the up- and downregulated genes for each of the three pairwise comparisons between stimulation groups. Comparison of GO terms’ raw *p*-values between all gene sets from all pairwise comparisons was done using heat maps of –log10 (*p*-values) made separately for biological processes (BP), molecular functions (MF) and cellular components (CC) categories using terms with raw *p*-values <1e-5 in any comparison for BP and < 1e-4 for MF and CC, due to their having fewer terms. A similar heat map was made for KEGG terms with raw *p*-values <1e-5 in any comparison, and also pathway maps showing both up- and downregulated genes were made for each pairwise comparison for any pathway with either up or downregulation with a *p*-value <1e-3 using the pathview package [27] (v 1.16.1), (data not shown).

## Results

### Flow cytometry

**Fig. 1:**
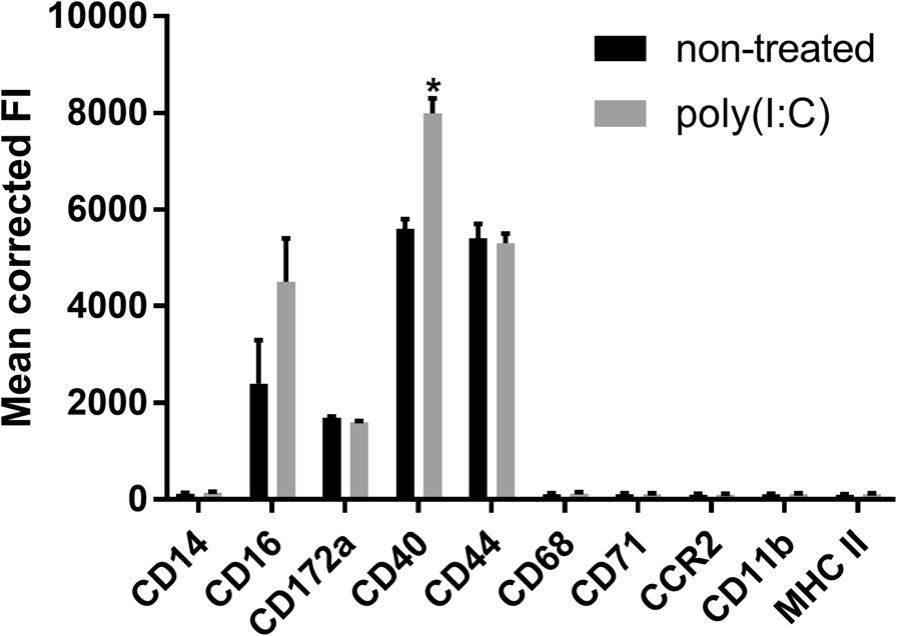
Flow cytometric analysis of Bomac cells. Bomac cells were labeled with antibodies against CD14, CD16, CD11b, CD172a, CD44, MHC II, CD40, CD68, CD71, CCR2 and using single color staining. Another set of cells were stimulated with p(I:C) as described in Material and Methods, and then similarly stained for flow cytometry. At least 100,000 events were acquired in FACS Calibur flow cytometer and analyzed in FlowJo software. Results are shown as mean fluorescence intensity (MFI). Six separate stainings for each group of cells were performed. * = *p* ≤ 0.05

Figure 1 shows the mean fluorescence results from a single colour staining of Bomac cells with antibodies against several surface markers of bovine macrophages. These results largely confirm what others [12] have already shown, with the exception of CD16. We observed expression of CD16, CD172a, CD40 and CD44. Only CD16 and CD40 (*p* ≤ 0.05) were upregulated following stimulation of the cells with p(I:C). Additionally, we scrutinized the phagocytic function of Bomac. As shown in Additional file 1, the Bomac can phogocytose *Staphylococcus aureus* labelled with FITC, albeit at very low level compared to freshly isolated bovine monocytes. However, the capability to phagocytose is not homogeneous, as majority of the cells did not engulf bacteria.

### Categorization and abundance of cDNA

**Fig. 2:**
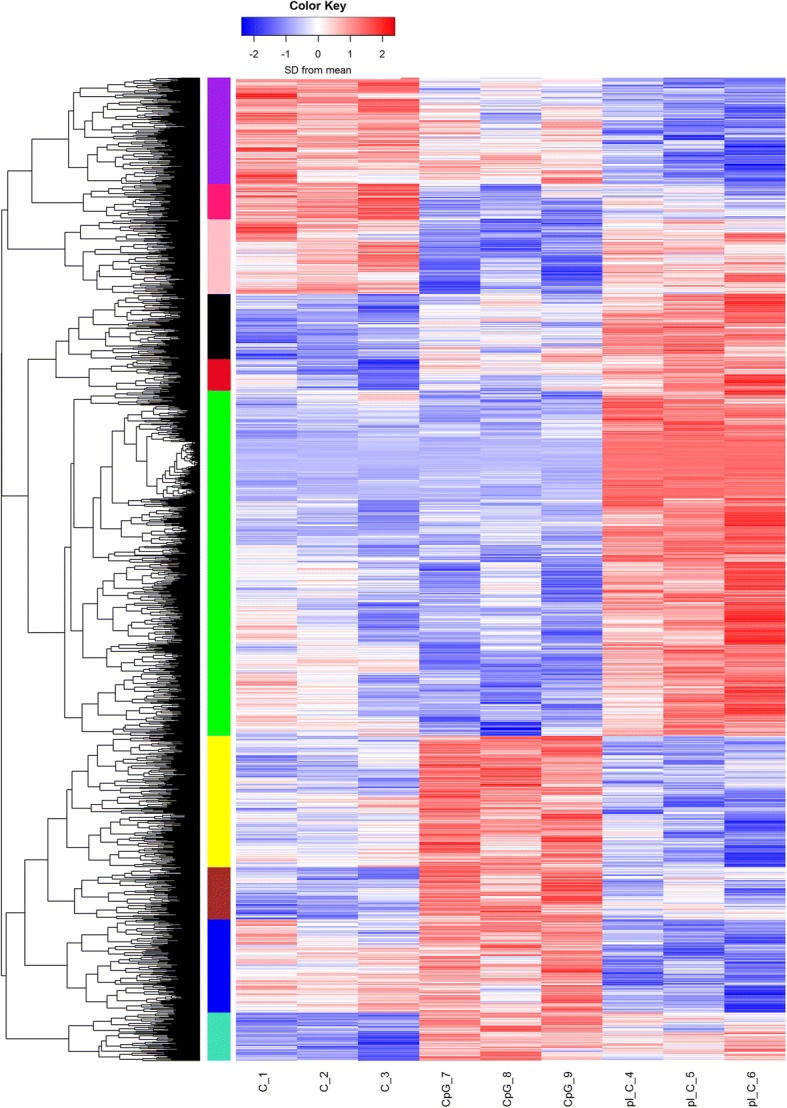
RNASeq Heat map representing 2245 genes differentially expressed in the Bomac cell line responding to stimulation with CpG DNA or poly(I:C). C_1, C_2 and C_3 are control experiments repeated three times. pI:C_4, pI:C_5 and pI:C_6 are Bomac cells stimulated with poly(I:C). CpG DNA_7, CpG DNA_8 and CpG DNA_9 Bomac cells stimulated with CpG DNA. All cells were incubated for 6 h, with each stimulation performed in three separate experiments

**Fig. 3:**
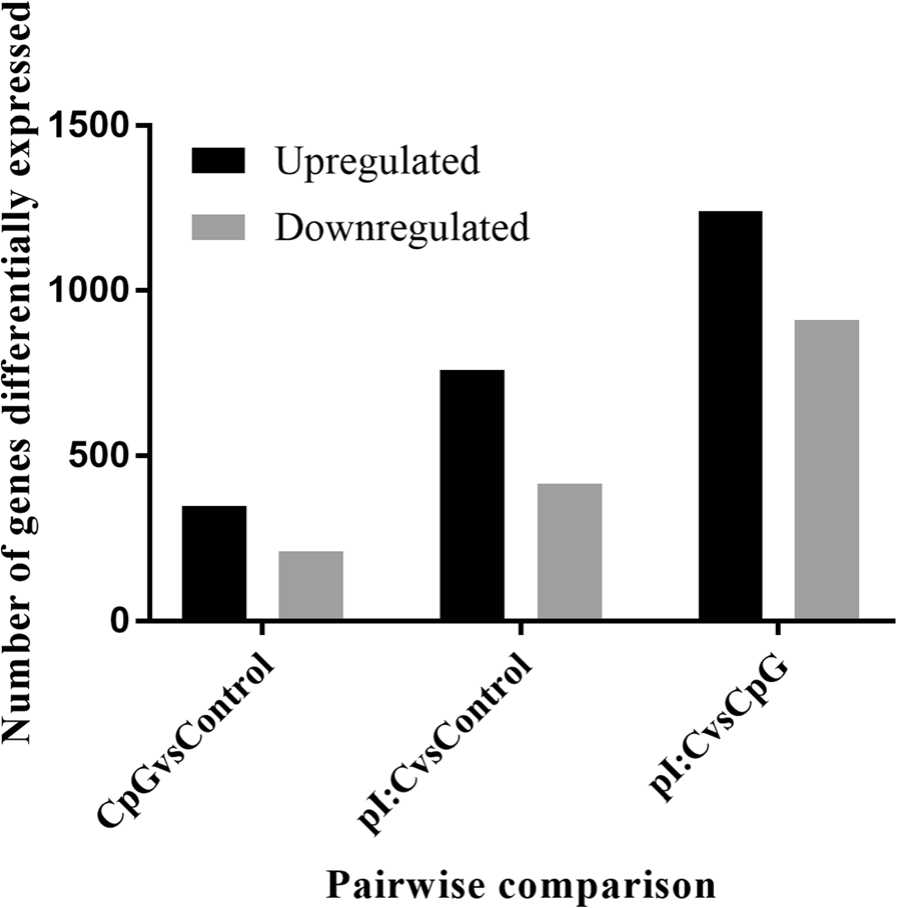
Pairwise comparison of differentially expressed genes in Bomac cells stimulated with CpG DNA or poly(I:C). The compared treatment groups are CpG DNA vs control, poly(I:C) vs control, and poly(I:C) vs CpG DNA

The statistics of categorization and abundance of sequence reads generated from 9 cDNA libraries used for differential gene expression analysis are shown in Table 1. A one-way ANOVA analysis revealed differences between the 2 test groups. 2245 genes out of 13,740 expressed in the data set had FDR *p*-values ≤0.05 (Fig. 2). The hierarchical clustering dendrogram of Euclidian distances between the genes (rows) was cut at height = 4, resulting in ten clusters. In comparison to control experiments, the number of genes with pairwise FDR *p ≤* 0.05 for CpG DNA-stimulated cells was 210 downregulated and 347 upregulated. In poly(I:C)-stimulated cells 414 genes were downregulated while 761 were upregulated. When the 2 treatment groups were compared, 911 genes were found to be downregulated whereas 1240 genes were upregulated (Fig. 3). Tables 2 and 3 show the top 10 upregulated and downregulated genes at 6 h post stimulation in both CpG DNA vs control and poly(I:C) vs control ranked by fold-change.

**Table 2:** Ranking of the top 10 upregulated and downregulated differentially expressed genes in the CpG DNA dataset. Ranking based on fold change

Gene	ID	Name	Fold change	*p* value	FDR
A					
TNFSF18	768,081	tumor necrosis factor superfamily member 18	5.3	6.9 × 10^−4^	2.4 × 10^− 2^
Slc26a10	506,076	solute carrier family 26 member 10	4.3	1.2 × 10^−3^	3.5 × 10^− 2^
NR4A1	528,390	nuclear receptor subfamily 4 group A member 1, transcript variant X3	3.1	9.7 × 10^−5^	6.2 × 10^− 3^
IGFLR1	617,594	IGF like family receptor 1	3.1	6.6 × 10^−5^	4.7 × 10^−3^
GSDMB	509,296	gasdermin B	2.5	6.4 × 10^−5^	4.6 × 10^−3^
LBH	616,148	limb bud and heart development	2.2	6.7 × 10^−14^	4.6 × 10^− 10^
LY6G5B	539,236	lymphocyte antigen 6 complex, locus G5B	2.2	1.4 × 10^−3^	3.9 × 10^− 2^
MYOZ2	540,487	myozenin 2, transcript variant X1	2.7	4.01 × 10^−5^	3.5 × 10^−3^
RRAD	505,165	Ras-related associated with diabetes	2.0	5.8 × 10^−5^	4.3 × 10^−3^
PLAU	281,983	plasminogen activator, urokinase receptor	1.7	4.6 × 10^−8^	2.1 × 10^−5^
B					
CYP1A1	282,870	cytochrome P450, subfamily I (aromatic compound-inducible), polypeptide 1, transcript variant X2	−2.8	4.3 × 10^−10^	6.6 × 10^−7^
CYP1B1	511,470	cytochrome P450, family 1, subfamily B, polypeptide 1	−2.4	2.0 × 10^− 10^	4.0 × 10^−7^
ID1	497,011	inhibitor of DNA binding 1, dominant negative helix-loop-helix protein	−2.4	3.5 × 10^− 16^	4.9 × 10^− 12^
GPR35	505,056	G protein-coupled receptor 35, transcript variant X7	− 2.4	2.4 × 10^−4^	1.2 × 10^− 2^
SLC25A34	515,553	solute carrier family 25 member 34	− 2.3	2.3 × 10^−5^	2.4 × 10^− 3^
EDN2	319,094	endothelin 2, transcript variant X1	− 2.1	3.4 × 10^−8^	1.7 × 10^−5^
ATOH8	616,225	atonal bHLH transcription factor 8, transcript variant X1	−2.1	5.1 × 10^− 9^	4.4 × 10^− 6^
ID3	538,690	inhibitor of DNA binding 3, dominant negative helix-loop-helix protein	−2.0	1.2 × 10^− 12^	5.8 × 10^− 9^
BDKRB1	532,119	bradykinin receptor B1	−1.9	1.1 × 10^−4^	6.7 × 10^− 3^

(A) upregulated, (B**)** downregulated. Data were analyzed in IPA (QIAGEN Inc., https://www.qiagenbioinformatics.com/products/ingenuity-pathway-analysis

**Table 3:** Ranking of the top 10 upregulated and downregulated differentially expressed genes in the poly(I:C) dataset. Ranking based on fold change

Gene	ID	Name	Fold change	*p* value	FDR
A					
TNFSF10	507,215	tumor necrosis factor superfamily member 10, transcript variant X1	3838.1	1.5 × 10^−17^	4.1 × 10^− 15^
IFIT2	527,528	interferon induced protein with tetratricopeptide repeats 2	2354.2	7.8 × 10^− 16^	1.5 × 10^− 13^
RSAD2	506,415	radical S-adenosyl methionine domain containing 2	2258.9	1.9 × 10^−28^	5.2 × 10^− 25^
IFI44L	508,347	interferon induced protein 44 like	2010.7	2.8 × 10^−15^	4.8 × 10^− 13^
OAS1^a^	347,699	2′,5′-oligoadenylate synthetase 1, 40/46 kDa	1591.7	9.7 × 10^− 23^	5.3 × 10^− 20^
IFI44	508,348	interferon induced protein 44, transcript variant X1	1254.3	1.8 × 10^− 13^	2.6 × 10^− 11^
ISG15	281,871	ISG15 ubiquitin-like modifier	1033.6	4.9 × 10^−30^	1.6 × 10^−26^
MX1	280,872	MX dynamin-like GTPase 1, transcript variant X2	967.8	1.8 × 10^−27^	3.6 × 10^− 24^
MX2	280,873	MX dynamin-like GTPase 2, transcript variant X1	895.4	4.0 × 10^− 20^	1.4 × 10^−17^
OAS2	529,660	2′-5′-oligoadenylate synthetase 2	807.5	8.7 × 10^− 12^	9.9 × 10^− 10^
B					
CYP1A1	282,870	cytochrome P450, subfamily I (aromatic compound-inducible), polypeptide 1, transcript variant X2	−3.8	8.1 × 10^− 12^	9.3 × 10^− 10^
EXOC3L2	539,328	exocyst complex component 3-like 2	− 3.7	1.2 × 10^− 4^	3.3 × 10^− 3^
USH1G	531,104	Usher syndrome 1G (autosomal recessive)	− 3.3	1.3 × 10^− 3^	2.1 × 10^− 2^
CYP26B1	540,868	cytochrome P450, family 26, subfamily B, polypeptide 1	− 3.1	1.9 × 10^− 3^	2.8 × 10^− 2^
SMIM17	100,849,034	small integral membrane protein 17, transcript variant X4	− 2.9	1.5 × 10^− 3^	2.3 × 10^− 2^
DAAM2	783,665	dishevelled associated activator of morphogenesis 2, transcript variant X1	− 2.8	1.1 × 10^− 3^	1.9 × 10^− 2^
TMEM52	617,403	transmembrane protein 52	− 2.7	3.5 × 10^− 3^	4.4 × 10^− 2^
CYP1B1	511,470	cytochrome P450, family 1, subfamily B, polypeptide 1	− 2.6	6.8 × 10^− 11^	6.9 × 10^− 9^
WASF3	540,674	WAS protein family member 3, transcript variant X4	−2.3	2.7 × 10^− 3^	3.1 × 10^− 2^
ATOH8	616,225	atonal bHLH transcription factor 8, transcript variant X1	− 2.2	9.5 × 10^− 10^	8.2 × 10^− 8^

(A) upregulated, (B) downregulated. Data were analyzed in IPA (QIAGEN Inc., https://www.qiagenbioinformatics.com/products/ingenuity-pathway-analysis

### Functional characterization of differentially expressed genes

#### KEGG pathways

**Fig. 4:**
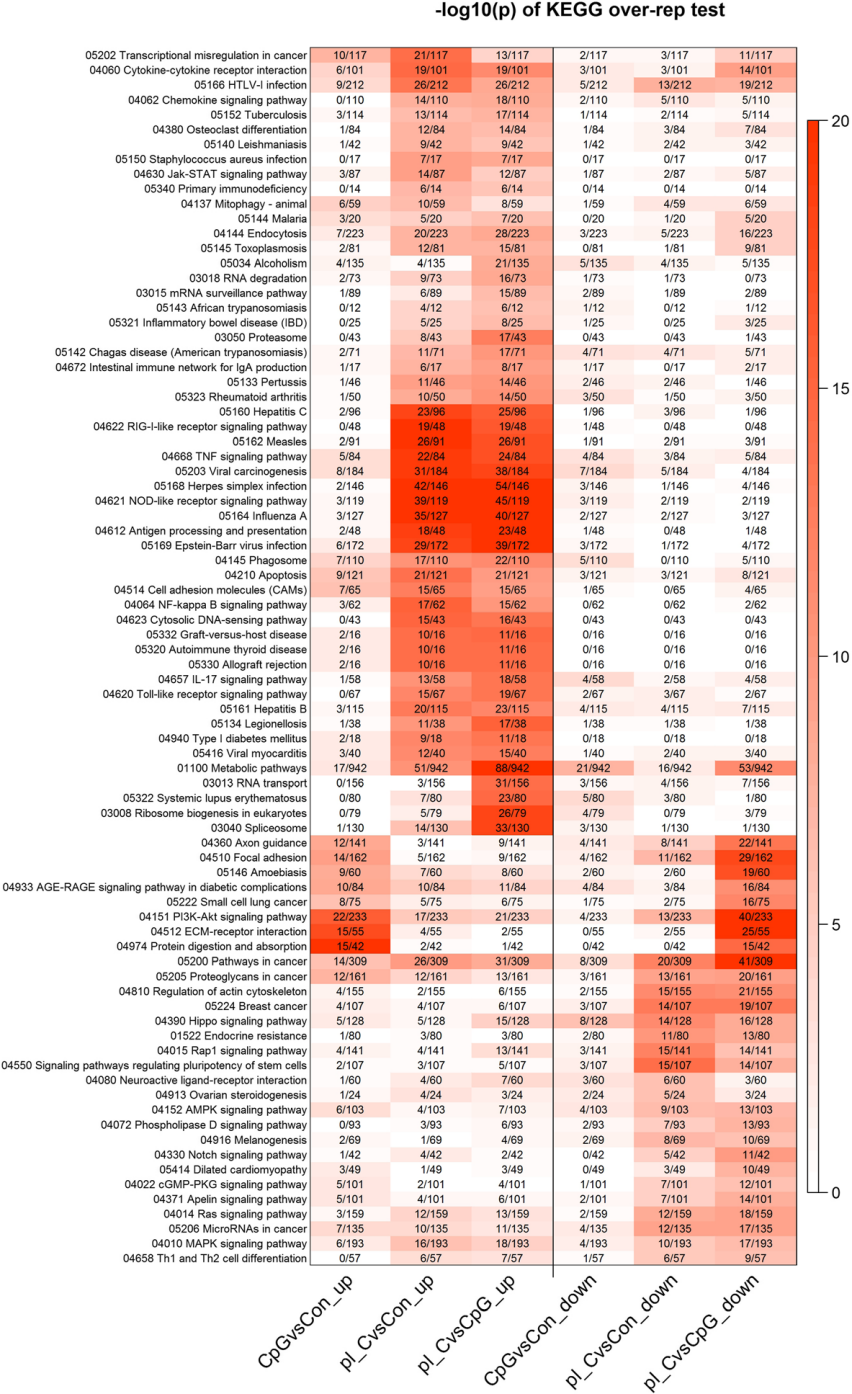
Representation of KEGG pathways. Each pathway is a row and each gene set is a column. The red color intensity shows the –log10 (raw *p*-value), i.e., a value of 6 = 1e-6. As the traditional FDR correction is not appropriate for KEGG or GO testing, we used an extremely low raw *p-*value to focus on the most highly significant pathways or terms. The numbers in the boxes show the number of genes in the pathway that are significantly up or downregulated, divided by the total number of genes in the pathway. CpG DNAvsCon_up = upregulated genes in the CpG DNA vs control comparison; pI_CvsCon_up = upregulated genes in the poly(I:C) vs control comparison; pI_CvsCpG DNA_up = upregulated genes in the poly(I:C) vs CpG DNA comparison; CpG DNAvsCon_down = downregulated genes in the CpG DNA vs control comparison; pI_CvsCon_down = downregulated genes in the poly(I:C) vs control comparison; pI_CvsCpG DNA_down = downregulated genes in the poly(I:C) vs CpG DNA comparison

There are 312 different KEGG pathways, of which 82 were highly overrepresented (raw *p*-value <1e-6) in at least 1 of the 3 pairwise gene sets (6 in total). Figure 4 shows a heat map comparing the 6 gene sets across these 82 pathways. Both poly(I:C) vs Con and poly(I:C) vs CpG DNA show similar up and downregulated pathways, indicating that poly(I:C) treatment induces a very different transcriptional profile in Bomac cells at 6 h post-stimulation than CpG DNA. In Table 4 (see also Additional file 3**)** an expansion is made on these data to show the top 5 KEGG pathways in each data set and the top 5 immune related pathways. In CpG DNA-stimulated cells, the pathways included axon guidance, focal adhesion, and protein digestion and absorption. Among immune related pathways, *PI3K-Akt signalling* and *Cytokine-cytokine receptor signalling* were overrepresented (Table 4Ab). Poly(I:C) stimulation induced upregulation of a number of genes in many different pathways. Upregulated pathways included various disease and immune-related pathways while various signalling pathways were downregulated. Among the highly represented ones were the *NOD-like receptor signaling pathway* (39 genes), *RIG-I-like receptor signaling pathway* (22 genes), and all pathways indicating activation of mechanisms associated with response to viral pathogens. An example of this can be observed in the three viral pathways, herpes simplex, influenza, and measles virus infection (Table 4Ba). Comparison of CpG DNA and poly(I:C) stimulations showed marked domination of pathways related to response to infection, induced by poly(I:C) stimulation (data not shown).

**Table 4:** Kyoto Encyclopedia of Genes and Genomes pathways generated from CpG DNA or poly(I:C)-stimulated Bomac cells tested at 6 h post stimulation vs control cells

A	KEGG ID	Pathway	N^a^	↑^b^	*p*-value	↓^c^	*p*-value
a	path:bta04974	Protein digestion and absorption	42	15	1.2 × 10^− 20^	0	1
	path:bta04512	ECM-receptor interaction	55	15	5.5 × 10^−18^	0	1
	path:bta04151	PI3K-Akt signaling pathway	233	22	1.3 × 10^−16^	4	4.3 × 10^−2^
	path:bta04510	Focal adhesion	162	14	5.5 × 10^−10^	4	1.8 × 10^−2^
	path:bta04360	Axon guidance	141	12	3.2 × 10^−09^	4	7.4 × 10^−3^
b	path:bta04151	PI3K-Akt signaling pathway	233	22	1.3 × 10^−16^	4	4.3 × 10^−2^
	path:bta04514	Cell adhesion molecules (CAMs)	65	7	7.2 × 10^−07^	1	2.7 × 10^−1^
	path:bta04210	Apoptosis	121	9	1.4 × 10^−06^	3	3.2 × 10^−2^
	path:bta03320	PPAR signaling pathway	44	6	1.7 × 10^−06^	1	2.1 × 10^−1^
	path:bta04060	Cytokine-cytokine receptor interaction	101	6	5.6 × 10^−05^	3	8.7 × 10^−3^
B							
a	path:bta04621	NOD-like receptor signaling pathway	119	39	3.9 × 10^−38^	2	2.4 × 10^−1^
	path:bta05168	Herpes simplex infection	146	42	4.9 × 10^−38^	1	6.9 × 10^−1^
	path:bta05164	Influenza A	127	35	6.0 × 10^−31^	2	2.8 × 10^−1^
	path:bta05162	Measles	91	26	2.8 × 10^−23^	2	1.8 × 10^−1^
	path:bta04622	RIG-I-like receptor signaling pathway	48	19	1.6 × 10^−20^	0	1
b	path:bta04621	NOD-like receptor signaling pathway	119	39	3.9 × 10^−38^	2	2.4 × 10^−1^
	path:bta04622	RIG-I-like receptor signaling pathway	48	19	1.6 × 10^−20^	0	1
	path:bta04668	TNF signaling pathway	84	22	1.1 × 10^− 19^	3	2.8 × 10^−2^
	path:bta04612	Antigen processing and presentation	48	18	2.4 × 10^− 19^	0	1
	path:bta04064	NF-kappa B signaling pathway	62	17	5.4 × 10^−16^	0	1

^a^number of genes in the pathway; ^b^number of genes up-regulated in the pathway; ^c^number of genes down-regulated in the pathway

(A) CpG DNA, (B) poly(I:C). (Aa and Ba) Represent top 5 pathways in CpG DNA and poly(I:C) datasets respectively; (Ab and Bb) represent top 5 pathways of immune related genes in the datasets. Gene names in each pathway are shown in the Additional file 3

#### Gene ontology

**Fig. 5:**
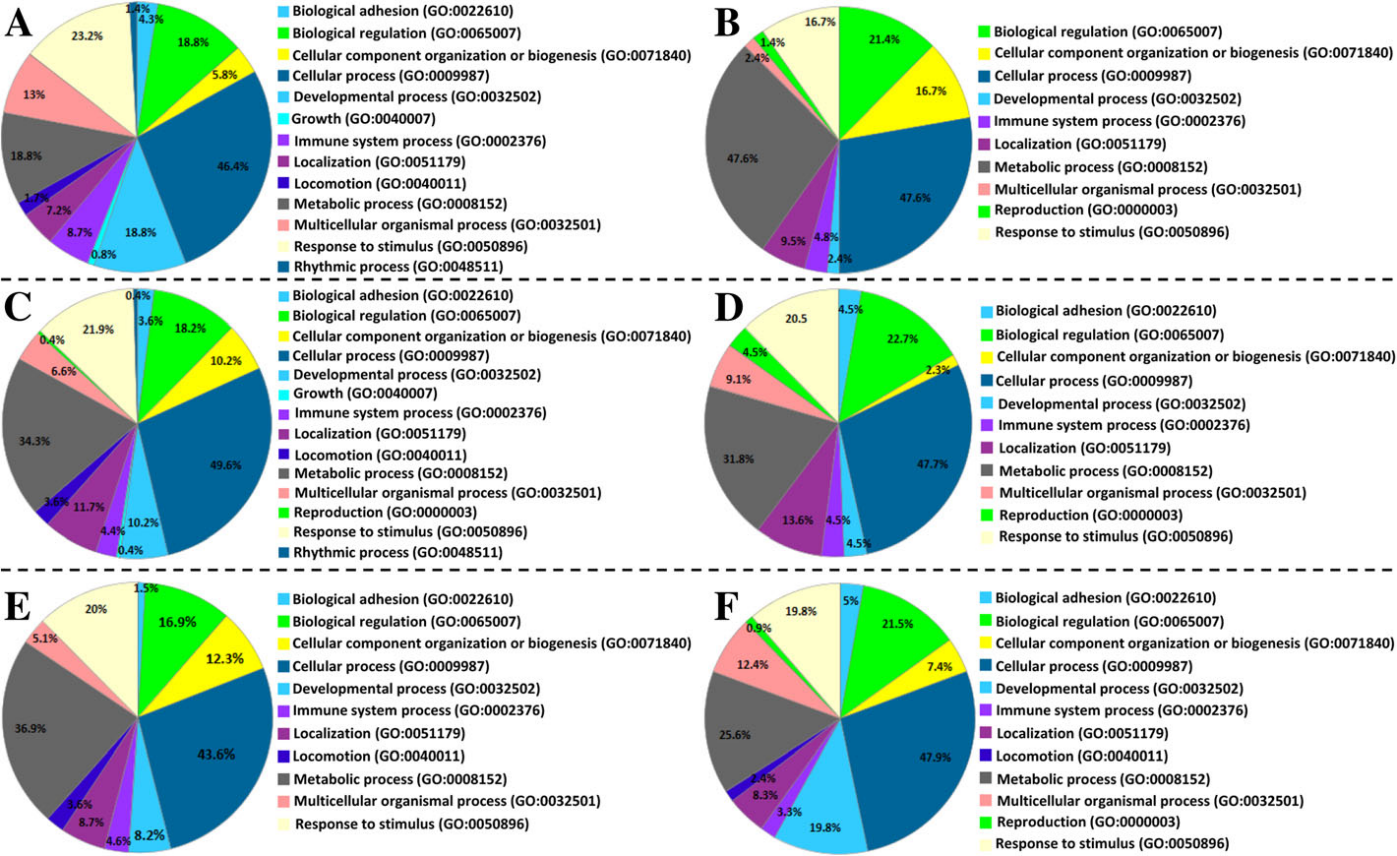
Gene ontology plotted by PANTHER (http://www.pantherdb.org/) showing biological processes for (**a**) CpG DNA vs Control upregulated genes; **b** CpG DNA vs Control downregulated genes; **c** poly(I:C) vs Control Upregulated genes; **d** poly(I:C) vs Control downregulated genes; **e** poly(I:C) vs CpG DNA upregulated genes; **f** poly(I:C) vs CpG DNA downregulated genes

**Fig. 6:**
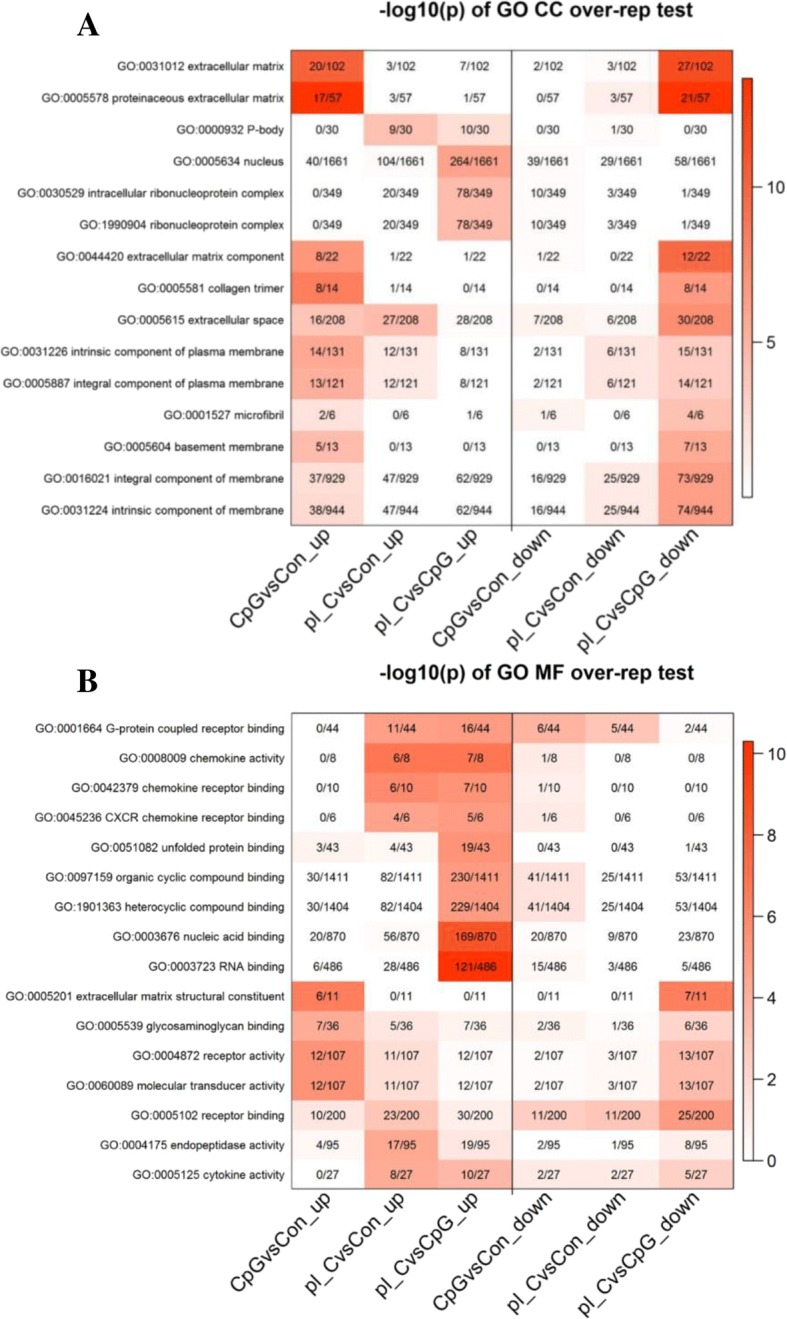
Charts presenting the Gene Ontology (GO) based classification of differentially expressed genes in Bomac cell line stimulated with CpG DNA or poly(I:C) to cellular components and molecular function processes. **a** Cellular Components (CC), **b** Molecular functions (MF). CpG DNAvsCon_up = upregulated genes in the CpG DNA vs control comparison; pI_CvsCon_up = upregulated genes in the poly(I:C) vs control comparison; pI_CvsCpG DNA_up = upregulated genes in the poly(I:C) vs CpG DNA comparison; CpG DNAvsCon_down = downregulated genes in the CpG DNA vs control comparison; pI_CvsCon_down = downregulated genes in the poly(I:C) vs control comparison; pI_CvsCpG DNA_down = downregulated genes in the poly(I:C) vs CpG DNA comparison

Gene ontology (GO) analysis was applied to these data to gain insight into the represented gene categories among the differentially expressed genes. Overall, 9490 terms were identified in the BP, 2629 terms in MF and 1273 terms in CC. A stringent raw *p* < 1e-5 was applied across the 6 gene sets, which reduced the number of terms to 76 BP terms, 13 MF terms and 11 CC terms. Due to a high number of GO terms in BP satisfying the condition raw *p* < 1e-5, the Panther functional classification tool was used to show only the main categories of GO terms. Figure 5 shows a pie chart representation of the Panther GO-Slim Bioprocess. In the bioprocesses, mainly cellular and metabolic processes were highly represented in all three pairwise comparisons. Immune system process upregulated genes represented 8.7, 4.4 and 4.6% of DE genes in CpG DNA-stimulated cells vs control, poly(I:C)-stimulated cells vs control and poly(I:C)-stimulated vs CpG DNA-stimulated cells, respectively. Downregulated immune system processes represented 4.8, 4.5 and 3.3% of DE in CpG DNA-stimulated cells vs control, poly(I:C)-stimulated cells vs control and poly(I:C)-stimulated vs CpG DNA-stimulated cells, respectively. The 13 MF and 11 CC terms are shown in Fig. 6a and b together with the number of genes involved. Additional file 4 further provides the statistics and gene identities found in the GO terms. Highly represented GO MF terms in poly(I:C) vs control and poly(I:C) vs CpG DNA comparisons included terms such as *G-protein coupled receptor binding*; *chemokine activity*; *chemokine receptor binding* and *CXCR chemokine receptor binding*. Additionally, in the poly(I:C) vs CpG DNA comparison, *nucleic acid binding* and *RNA binding* were highly represented. GO CC terms were represented mainly by *extracellular matrix component* genes in CpG DNA vs control and poly(I:C) vs CpG DNA comparisons both in upregulation and downregulation, respectively.

#### Ingenuity pathway analysis (IPA)

Qiagen’s Ingenuity Pathway Analysis was employed to further scrutinize the DE genes https://www.qiagenbioinformatics.com/products/ingenuity-pathway-analysis [28]. The genes were mapped to human/mouse/rat genome for homology. There were 38 canonical pathways generated from the DE genes in CpG DNA vs control dataset and 25 from the DE genes in poly(I:C) vs control dataset. Table 5 shows the top 5 canonical pathways in both datasets. Interestingly, only 2 immune related canonical pathways were represented among the top 5 canonical pathways in the CpG DNA vs control dataset. In the *Nur77 Signalling in T Lymphocytes* pathway *HDACG*, *HLA-DMB*, *NR4A1* were upregulated and *CD3G* was downregulated. In the second pathway, *calcium-induced T Lymphocyte Apoptosis* only *HLA-DMB* and *NR4A1* were upregulated. The next highest ranking (16th) canonical pathway in the CpG DNA dataset was *CCL5 signalling in macrophages* with three cardinal genes (*CD3G, GNB3, PRKCB*) of which *CD3G* and *PRKCB* being downregulated (− 1.8 and − 1.6-fold change, FDR *p-*value 1.85 × 10^− 2^ and 1.08 × 10^− 1^, respectively) and *GNB3* upregulated (2-fold change, FDR *p*-value 1.23 × 10^− 1^). All the 25 canonical pathways generated in the poly(I:C) dataset were related to cellular immune function. The topmost canonical pathway was *interferon signalling* containing important transcription factor genes such as *NFκBIA* (+ 2.2-fold change, FDR *p-*value 1.5 × 10^− 6^) and transcription regulators such as *STAT1* (+ 3.2-fold change, FDR *p-*value 6.1 × 10^− 17^) and *STAT2* (1.9-fold change, FDR *p-*value 3.1 × 10^− 11^).

**Table 5:** Top 5 Canonical pathways generated by Ingenuity Pathway Analysis (IPA) of differentially expressed genes in Bomac cells stimulated with PAMPs (A) CpG DNA vs control, (B) poly(I:C) vs control. https://www.qiagenbioinformatics.com/products/ingenuity-pathway-analysis

	Canonical Pathway	*p*-Value	Ratio	Molecules
A	Sperm Motility	3.6 × 10^−4^	4.8 × 10^−2^	CNGA, GUY1B3, MST1R, PDE4B, PLA2G12B, PRKCB
	Nur77 Signaling in T Lymphocytes	9.9 × 10^−4^	6.8 × 10^−2^	CD3G, HDACG, HLA-DMB, NR4A1
	Xenobiotic Metabolism Signaling	1.5 × 10^−3^	2.8 × 10^−2^	CYP1A1, CYP1B1, FTL, HMOXX1, HS3ST2, MAP3K13, MAP3K14
	Calcium-induced T Lymphocyte Apoptosis	1.5 × 10^−3^	6.1 × 10^−2^	CD3G, HLA-DMB, NRA41, PRKCB,
	Coagulation System	2.3 × 10^−3^	8.6 × 10^−2^	BDKRB1, PLAU, PLAUR,
B	Interferon Signaling	2.1 × 10^−17^	4.17 × ^10–1^	SOCS1, IFIT3, OAS1, MX1, IRF9, IFI35, PSMB8, TAP1, IRF1, ISG15, IFIT1, STAT2, IFI6, STAT1, IFITM1
	Activation of IRF by Cytosolic Pattern Recognition Receptors	2.3 × 10^− 13^	2.42 × 10^− 1^	DHX58, NFKBIE, ZBP1, IRF9, IRF3, ADAR, ISG15, IFIH1, IRF7, NFKBIA, CD40, DDX58, STAT2, IFIT2, STAT1
	Role of Pattern Recognition Receptors in Recognition of Bacteria and Viruses	4.6 × 10^− 10^	1.24 × 10^− 1^	CXCL8, OAS1, C3, OAS2, CCL5, IRF3, RNASEL, TLR2, IFIH1, IRF7, DDX58, TGFB3, PRKCH, IRS2, EIF2AK2, RIPK2, C3AR1
	Death Receptor Signaling	1.1 × 10^−9^	1.52 × 10^−1^	GAS2, DAXX, NFKBIA, PARP10, NFKBIE, ZC3HAV1, TNFSF10, PARP12, PARP3, CASP8, BIRC3, PARP9, CASP7, PARP14
	Antigen Presentation Pathway	1.9 × 10^−8^	2.37 × 10^−1^	B2M, PSMB9, NLRC5, HLA-B, HLA-DMB, CIITA, PSMB8, TAP1, TAP2

**Fig. 7:**
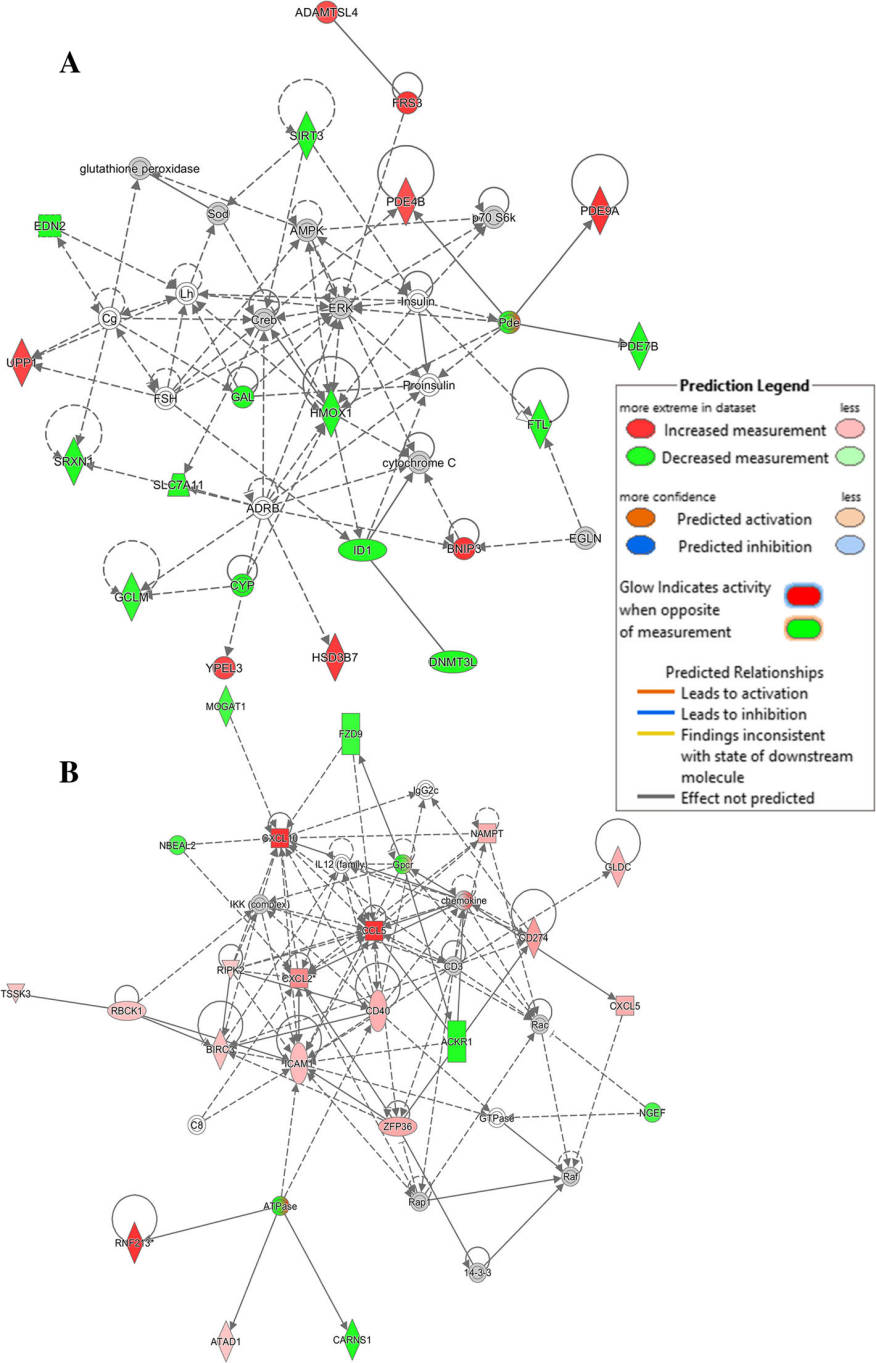
The top networks identified in differentially expressed genes in CpG DNA and poly(I:C)-stimulated Bomac cell line after 6 h stimulation, based on Ingenuity Pathway Analysis (IPA, Qiagen) https://www.qiagenbioinformatics.com/products/ingenuity-pathway-analysis. **a** CpG DNA-stimulated cells, Hematological Disease, Respiratory Disease, Organismal Injury and Abnormalities network, **b** poly(I:C)-stimulated cell, Cell-To-Cell Signaling and Interaction, Cellular Movement, Hematological System Development and Function

To show relevant relationships between molecules in the 2 datasets, core analysis in IPA was performed to search for networks. In total, 11 networks were identified in the CpG DNA dataset and 81 in the poly(I:C) dataset. The top 5 in each dataset are shown in Table 6 and the topmost networks are shown in Fig. 7. No immune-related network was represented among the top 5 of the networks in the CpG DNA vs control dataset, whereas in the poly(I:C) dataset, 4 out of 5 networks were immune related. The top network in CpG DNA-stimulated cells, *Hematological Disease, Respiratory Disease, Organismal Injury and Abnormalities* contained a total of 19 genes found in the dataset, 8 were upregulated and the remaining 12 were downregulated (Fig. 7a). In poly(I:C)-stimulated cells, the top network was *Cell-To-Cell Signaling and Interaction, Cellular Movement, Hematological System Development and Function* containing 22 genes identified in the dataset out of which 6 were downregulated and the remaining were upregulated (Fig. 7b)**.**

**Table 6:** Top Networks generated from DE genes in Bomac cell line treated with CpG DNA or poly(I:C) for 6 h. (A) CpG DNA, (B) poly(I:C). Data were analyzed in IPA (QIAGEN Inc., https://www.qiagenbioinformatics.com/products/ingenuity-pathway-analysis

	Associated Network Functions	Score
A	Hematological Disease, Respiratory Disease, Organismal Injury and Abnormalities	34
	Organismal Functions, Hereditary Disorder, Neurological Disease	32
	Cellular Development, Connective Tissue Development and Function, Tissue Development	27
	Cellular Assembly and Organization, Cellular Function and Maintenance, Molecular Transport	27
	Cell Cycle, Embryonic Development, Cancer	27
B	Cell-To-Cell Signaling and Interaction, Cellular Movement, Hematological System Development and Function	33
	Cell Death and Survival, Cancer, Hematological Disease	33
	Protein Synthesis, Lipid Metabolism, Small Molecule Biochemistry	31
	Cell Death and Survival, Infectious Diseases, Cancer	29
	Infectious Diseases, Antimicrobial Response, Inflammatory Response	23

**Fig. 8:**
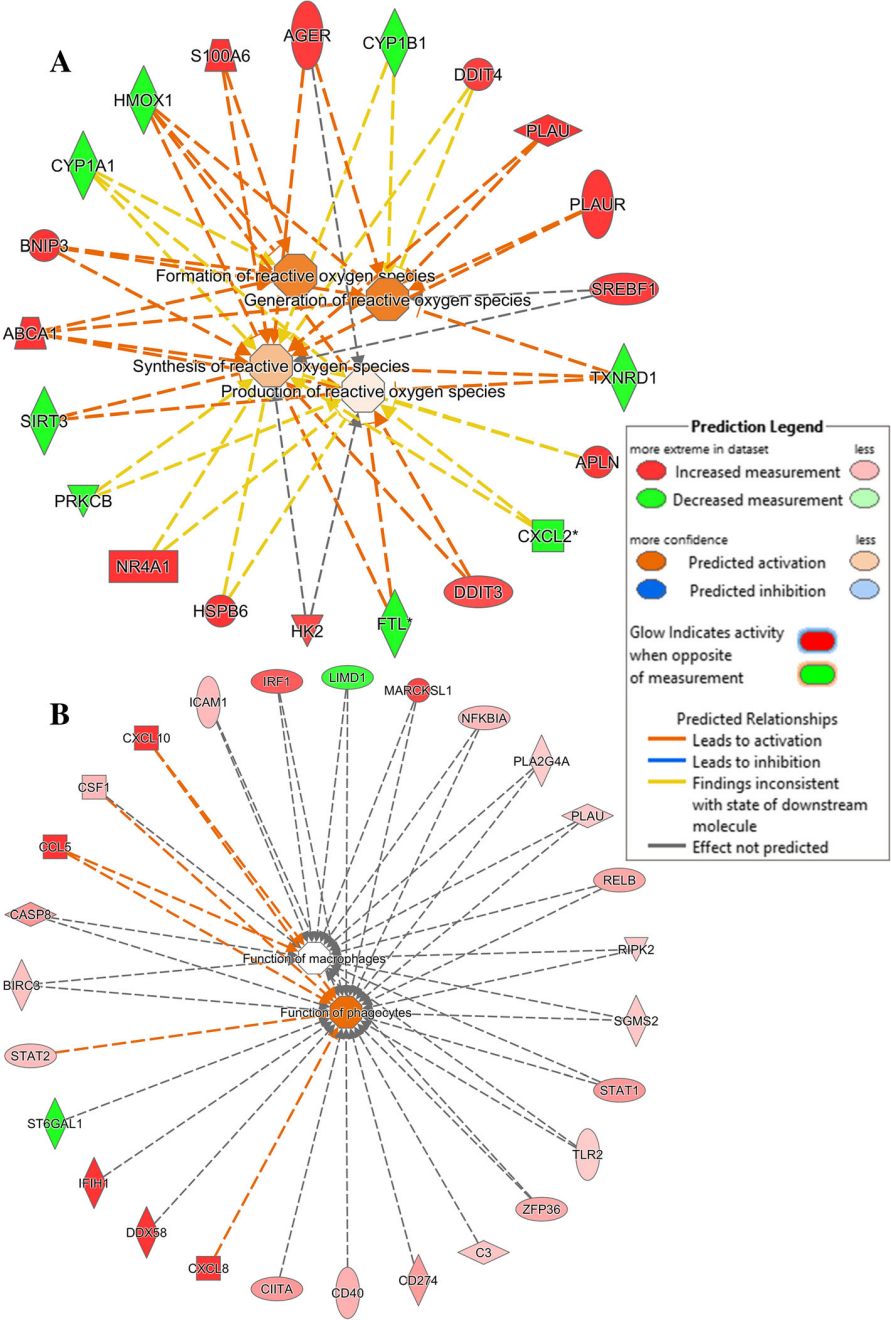
Functional networks identified in differentially expressed genes in Bomac cells treated with CpG DNA or poly(I:C) upon analysis with Ingenuity Pathway Analysis (IPA). **a** CpG DNA-stimulated cells, Free Radical Scavenging molecular function network in bovine MΦ, **b** poly(I:C)-stimulated cells, Cellular Function and Maintenance network in bovine MΦ. https://www.qiagenbioinformatics.com/products/ingenuity-pathway-analysis

Further, the molecular and cellular function were closely examined in the datasets. Table 7 shows the top 5 significant functional networks in the two datasets. The topmost function in CpG DNA-stimulated cells was the free radical scavenging (*p*-value 1.45 × 10^− 8^) containing 21 genes (e.g., *NR4A1, +* 3.2-fold change FDR *p-*value 6 × 10^− 3^, *DDIT4 +* 1.67-fold change FDR *p-*value, 5.9 × 10^− 4^*, PLAUR +* 1.76-fold change, FDR *p-*value 2.1 × 10^− 5^). Figure 8a shows a network of molecules responsible for *formation of reactive oxygen species*, *generation of reactive oxygen species*, *synthesis of reactive oxygen species* and *production of reactive oxygen species* in macrophages. The *cellular function and maintenance* was the most significantly represented function group in poly(I:C)-stimulated cells (Table 7B, *p-*value 2.64 × 10^− 16^) with 110 molecules (e.g., *DDX58,* + 18 fold-change *p*-value 1.1 × 10^− 22^*, CCL5* + 96 fold-change *p*-value 4.5 × 10^− 11^*, IRF1* + 4.8-fold change *p*-value 5.13 × 10^− 16^) responsible for the *function of macrophages* (Fig. 8b).

**Table 7:** Common and unique Molecular and Cellular Functions pathways within the top 5 pathways identified in the differentially expressed genes in CpG or pI:C-treated Bomac cells. Data were analyzed in IPA (QIAGEN Inc., https://www.qiagenbioinformatics.com/products/ingenuity-pathway-analysis

	Name	*p*-value	#Molecules
Common			
pI:C	Cellular Function and Maintenance	1.5 × 10^−4^ - 2.6 × 10^−16^	110
CpG	Cellular Function and Maintenance	7.1 × 10^−3^ - 2.4 × 10^−6^	62
pI:C	Cell Death and Survival	2.1 × 10^−4^ - 4.3 × 10^−16^	149
CpG	Cell Death and Survival	7.5 × 10^−3^ - 9.4 × 10^−7^	58
pI:C	Cellular Development	2.6 × 10^−4^ - 4.8 × 10^− 11^	134
CpG	Cellular Development	7.0 × 10^−3^ - 7.3 × 10^−6^	69
Unique			
CpG	Free Radical Scavenging	5.8 × 10^−4^ - 1.4 × 10^−8^	21
CpG	Cell Morphology	7.3 × 10^−3^ - 7.3 × 10^−6^	50
pI:C	Cell Signaling	1.2 × 10^−5^ - 4.9 × 10^−15^	38
pI:C	Cellular Growth and Proliferation	2.1 × 10^−4^ - 4.8 × 10^− 11^	120

**Fig. 9:**
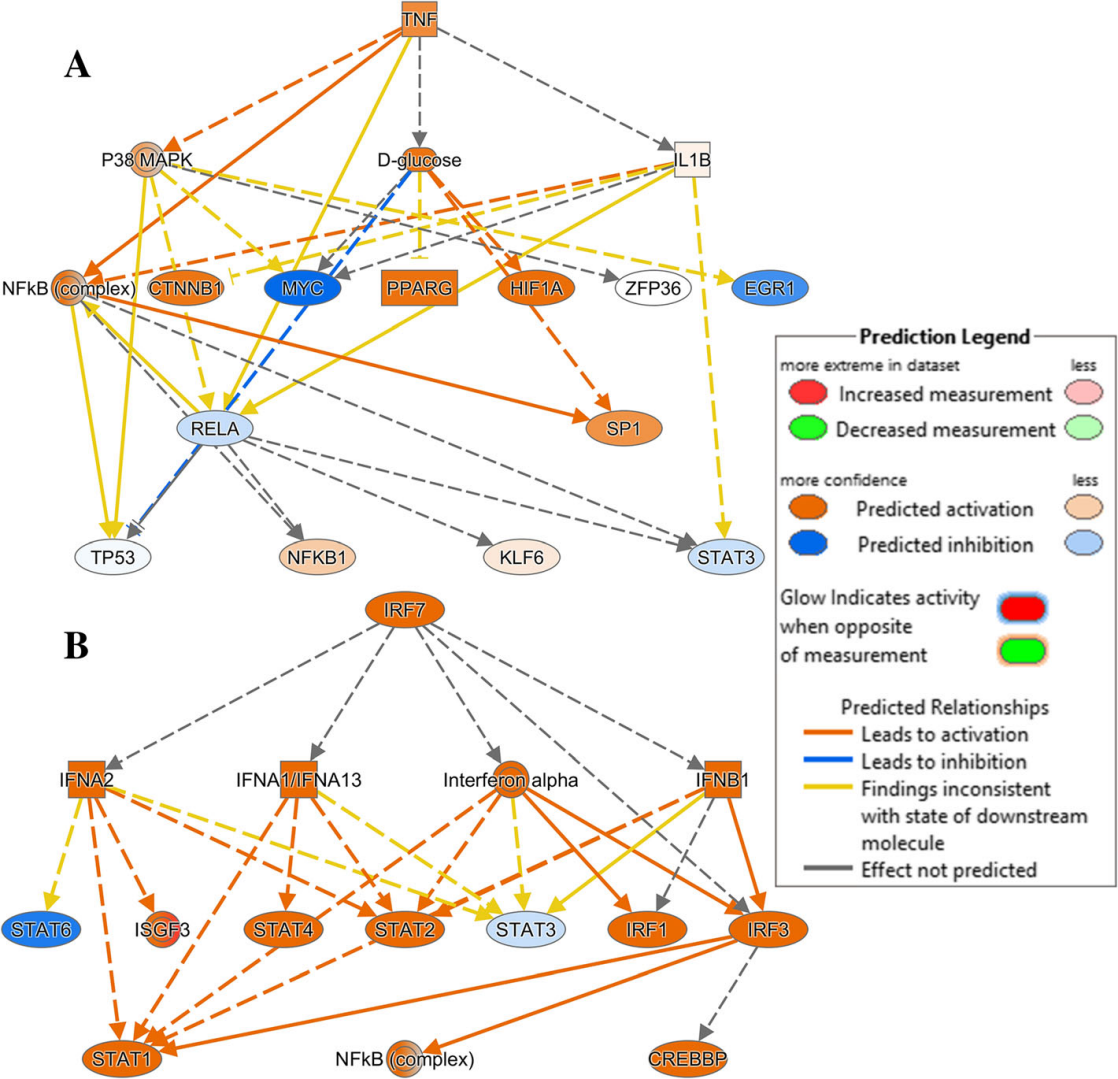
Mechanistic networks generated from differentially expressed genes in datasets derived from Bomac cells stimulated with CpG DNA or poly(I:C) for 6 h. **a** CpG DNA, TNF upstream regulator, **b** poly(I:C), IRF7 upstream regulator. The mechanistic networks were created with the use of IPA (QIAGEN Inc., https://www.qiagenbioinformatics.com/products/ingenuity-pathway-analysis

Upstream regulator analysis was performed to identify upstream regulators that might have influenced the gene expression changes observed in the two datasets. 1334 and 1721 molecules in CpG DNA and poly(I:C)-stimulated cells, respectively, were identified of which the top 5 regulators for each data set are listed in Table 8. The top most regulator in the CpG DNA dataset was the tumor necrosis factor (*TNF*) (upregulated, FDR *p*-value 2.3 × 10^− 10^) and it affected 40 target molecules, while the poly(I:C) dataset revealed *IRF7* (upregulated, FDR *p-*value 1.38 × 10^− 57^) as the topmost regulator with 57 target molecules **(**Additional file 3). Mechanistic networks involving *TNF* and *IRF7* to show a plausible set of connected upstream regulators that have contributed to the gene expression changes observed in the CpG DNA and poly(I:C) datasets are shown in Fig. 9.

**Table 8:** Upstream Regulators identified in CpG DNA and poly(I:C) treated Bomac cell line following 6 h of stimulation. (A) CpG DNA, (B) poly(I:C). Data were analyzed in IPA (QIAGEN Inc., https://www.qiagenbioinformatics.com/products/ingenuity-pathway-analysis

	Upstream Regulator	*p*-value of overlap
A	TNF	2.31 × 10^−10^
	ADRB	9.58 × 10^−10^
	D-glucose	9.20 × 10^−09^
	lipopolysaccharide	1.71 × 10^−08^
	dexamethasone	2.11 × 10^− 08^
B	IRF7	1.38 × 10^−57^
	poly rI:rC-RNA	1.08 × 10^−46^
	IFNL1	2.53 × 10^− 46^
	IFNA2	8.27 × 10^−44^
	Interferon alpha	3.40 × 10^−43^

**Fig. 10:**
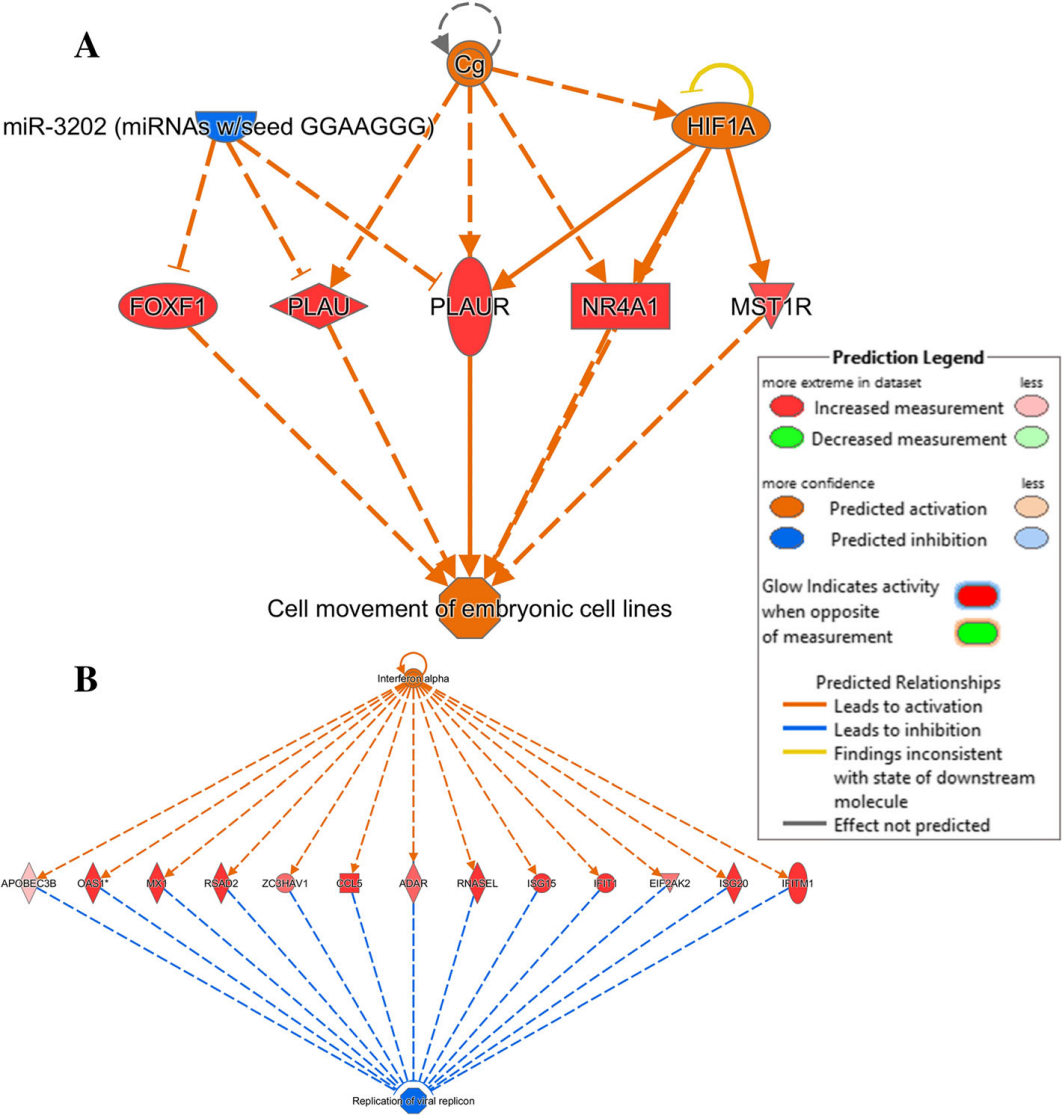
Top 5 regulator effect networks from each of the two datasets generated by Ingenuity Pathways. Bomac cells were treated with CpG DNA (**a**) or poly(I:C) (**b**) for 6 h. https://www.qiagenbioinformatics.com/products/ingenuity-pathway-analysis

Finally, the regulator effects in both datasets were addressed. The most significant regulator effects predicted for CpG DNA and poly(I:C)-stimulated cells are shown in Table 9. Three regulators *Cg*, *HIF1A*, miR-3202 (miRNAs w/seed GGAAGGG) in CpG DNA-stimulated cells had the highest consistency score and regulated 5 molecules (*FOXF1, MIST1R, NR4A1, PLAU, PLUAR)* responsible for cell movement of embryonic cells (Fig. 10a). All these genes were significantly upregulated in the dataset. One of the regulators was a miRNA that apparently regulates *FOXF1*, a transcription regulator. Poly(I:C)-stimulated cells revealed IFN-α as the main regulator with the highest consistency score, regulating at least 13 targets (*ADAR, APOBEC3B, CCL5, EIF2AK2, IFIT1, IFITM1, ISG15, ISG20, MX1, OAS1, RNASEL, RSAD2, ZC3HAV1*) (Fig. 10b), all of them participating in inhibition of replication of viral replicons. Similar IPA analysis was done on the dataset comparing poly(I:C) to CpG DNA-induced changes. Data can be found in Additional files 4, 5, 6 and 7.

**Table 9:** Top Regulator Effect Networks generated from CpG DNA and poly(I:C)-stimulated Bomac cell line after 6 h of stimulation. **(A)** CpG DNA, **(B)** poly(I:C). Data were analyzed in IPA (QIAGEN Inc., https://www.qiagenbioinformatics.com/products/ingenuity-pathway-analysis

	Regulators	Diseases & Functions	Consistency Score
A	Cg,HIF1A,miR-3202 (miRNAs w/seed GGAAGGG)	cell movement of embryonic cell lines	4.025
	BMP6,Ins1,MYC	lymphoproliferative disorder	3.328
	ERG,RXRA	Growth Failure	2.121
	miR-4525 (and other miRNAs w/seed GGGGGAU)	Growth Failure	−4.082
	IGF1	Growth Failure	−5.367
B	Interferon alpha	Replication of viral replicon	3.606
	Interferon alpha	Viral life cycle	3.606
	lipopolysaccharide	Replication of viral replicon	3.464
	PRL	Immune response of cells	3.357
	IFNA2	Replication of viral replicon	3.317

## Discussion

RNASeq and systems biology tools were applied to unravel the transcriptomic landscape in vitro MΦ stimulated with CpG DNA or poly(I:C) as a model for bacterial and viral pathogen-associated molecular patterns, respectively. Using the data derived from gene expression analysis, IPA, KEGG and GO were used to reconstruct cardinal networks, pathways, and cellular function groups of genes differentially expressed in the Bomac cell line stimulated with CpG DNA or poly(I:C). It should be noted that in IPA analyses, the genes were mapped to human/mouse/rat genome for homology. The Bomac cell line, developed from bovine peritoneal macrophages by Stabel and Stabel in 1995 [11], was previously tested as an infection model for *Mycoplasma bovis* to investigate cellular mechanisms involved in mycoplasma–Bomac cell interaction. Most recently both BVDV-infected and BVDV-free Bomac cells were tested for mycoplasmal uptake, growth in co-culture, viability, cytotoxicity and induction of apoptosis after infection with *M. bovis*. Cytotoxicity was increased after infection of BVDV-free cells with *M. bovis*, while apoptotic cell death was induced by *M. bovis* in both cell lines [13].

Although CpG DNA and poly(I:C) are well known for their adjuvant effects in stimulating immune responses, our results show that gene expression induced by each of these PAMPs in a MΦ cell line has common profiles and profiles that are different. For example, among DE genes 347 were upregulated and 210 were down regulated in Bomac stimulated with CpG DNA compared to 761 upregulated and 414 downregulated in poly(I:C)-stimulated cells at 6 h. There were more upregulated genes in the poly(I:C)-stimulated cells than CpG DNA-stimulated cells and the FDR adjusted *p*-values of upregulated genes were significantly higher compared to downregulated genes. The latter part of this observation was consistent with observations by others using monocyte-derived MΦ infected with MAP [4, 7]. Further, unlike CpG DNA-stimulated cells, poly(I:C) stimulation of Bomac cells produced an expression profile that may be expected following infection with RNA viruses such as influenza or measles virus. Responses to these two viruses involve activation of transcription factor genes such as *STAT1, STAT2* and *NFκB1*, which were upregulated in the Bomac cells stimulated with poly(I:C). Most canonical pathways represented within the DE genes in CpG DNA-stimulated cells were not related to immune functions, whereas the majority of canonical pathways in poly(I:C)-stimulated cells were related to immune functions. Stimulation of Bomac cells with CpG DNA highly affected the genes that regulate the phagocytic function of MΦ in contrast to stimulation with poly(I:C). This also confirms the importance to work with bovine cells in the absence of contaminations with ruminant pestiviruses, as BVDV was reported to inhibit the phagocytic activity of MΦs and neutrophils [12, 29]. However, immunological studies with these cells should be cautious, as these cells appear to poorly represent a lineage-specific phenotype [12].

Common transcriptomic profiles between CpG DNA-stimulated and poly(I:C)-stimulated cells were: (i) upstream and regulator effects, both stimulations induced immune related upstream regulators; (ii) in the top expressed genes, both stimuli induced high expression of the tumor necrosis factor family molecules albeit at different levels. CpG DNA induced expression of *TNFSF18* (+ 5.3-fold change, FDR *p*-value 2.4 × 10^− 2^). *TNFSF18* a ligand for TNFRSF18 expressed on the cell surface of regulatory T cells [30]. TNFSF18 is also released upon activation of DCs with CpG DNA [31]. In that report, plasmacytoid dendritic cells (pDCs) activated by CpG DNA promoted NK cell cytotoxicity and IFNγ production through type I IFNs and GITRL. Poly(I:C) induced expression of *TNFSF10* (> 3500-fold change, FDR *p-*value 1.5 × 10^− 17^) in this dataset. It is expressed at significant levels in most tissues including MΦs [32], and activates cysteine-type endopeptidase activity involved in apoptosis of transformed or tumor cells but does not kill normal cells [33]. However, our result is in contrast to that obtained by Casey et al. [7] who found that *TNFSF18* was downregulated at 6 h post infection of monocyte-derived bovine MΦs with *Mycobacterium avium* subspecies *paratuberculosis*. Although the analytical technique used was the same as ours, in their case, cells were derived from live animals compared to our bovine MΦ cell line. The most downregulated gene in both treatments was *CYP1A1* (*cytochrome P450, subfamily I (aromatic compound-inducible), polypeptide 1, transcript variant X2*). We could not find a specific role described for *CYP1A1* among the immune functions of MΦs, but assume that the gene product of *CYP1A1* could be involved in other cellular processes. According to a review by Androutsopoulos et al., [34] CYP1A1 plays an important role in the detoxication of environmental carcinogens, as well as in the metabolic activation of dietary compounds with cancer preventative activity. The KEGG pathways overrepresentation results were very similar to those generated in the IPA pathways analyses, both reporting a marked polarization of the response of MΦ to poly(I:C) towards antiviral activity.

In contrast to stimulation of Bomac with CpG DNA, cells stimulated with poly(I:C) had the highest number of DE genes suited to antiviral response of MΦs. *IFIT2* (> 2300-fold change, FDR *p*-value 1.5 × 10^− 13^) induced by interferon and regulated by *IRF1* (+ 4.8-fold change, FDR *p*-value 5.13 × 10^− 16^) gives rise to gene products that have antiviral functions [35]. *IRF1* further regulates *RSAD2* (> 2258.2-fold change, FDR *p*-value 5.2 × 10^− 25^), which has been reported to be highly expressed during viral infections [36, 37]. *IFI44L* and *IFI44* were upregulated (fold change + 2010.7 and + 1254.3, FDR *p* value 4.8 × 10^− 13^ and 2.6 × 10^− 11^, respectively) and play a role in immune responses against viruses, although the precise mechanism has not been described. The remaining highly expressed genes *OAS1, ISG15, MX1, MX2* and *OAS2* are well characterized antiviral response genes in many animal species [35, 38–40]. A study in mouse central nervous system tissue by Pomeranz et al. [41] showed a very similar profile to ours of highly expressed antiviral genes after infection with an RNA virus. In regard to highly expressed genes in the CpG DNA-stimulated cells, genes such as *SLC26A10, IGFLR1, GSDMB, LBH, MYOZ2* and *RRAD* have no clearly described role in immune responses of MΦs. *GSDMB* appears to be associated with asthma and certain types of autoimmune diseases such as, type 1 diabetes, inflammatory bowel disease, and rheumatoid arthritis [42, 43]. Ekwall et al. identified *LBH* as a candidate gene in rheumatoid arthritis and it was expressed in the synovial lining layer in such patients [44]. *NR4A1* is a nuclear transcription factor that belongs to the *nur77 signalling in T lymphocytes* and *calcium-induced T lymphocyte apoptosis* canonical pathways. These pathways were both represented as top canonical pathways in the CpG DNA dataset. *NR4A1* is expressed in MΦs in response to pro-inflammatory stimuli [45]. *PLAU* participates in the *coagulation system* canonical pathway represented as the 5th ranking canonical pathway in the CpG DNA data set. Expression of *PLAU* appears to be regulated by *TGFB1* [46]. Such a pattern of gene expression provides evidence for the advantage of whole genome analysis because it allows the detection of other genes highly expressed that might not directly relate to the immune function of MΦ but rather account for other functions of cells during an immune response. This provides an opportunity to further study functions of such genes in order to fully understand regulation of immune processes in which bovine MΦs may be engaged. An interesting finding was the occurrence of *PRL* (encoding prolactin) as a regulator in poly(I:C)-stimulated cells. Although prolactin is widely known for its essential role in lactation, it appears to have important roles in immune processes, indicating that MΦ may have a prolactin regulated function during reproduction [47, 48].

Close examination of the canonical pathways based on IPA analysis of poly(I:C) dataset shows that majority of these pathways are directly involved in immune response of MΦs. In the interferon pathway, at least five genes were interferon-inducible (*IFIT3, IFI35, IFIT1, IFI6, IFITM1*), and two genes were interferon regulatory genes (*IRF1, IRF9*) and the remaining 8 genes (*SOCS1, OAS1, MX1, PSMB8, TAP1, ISG15, STAT2, STAT1*) have activity highly regulated by interferon. Signaling through TLR3 upon stimulation with synthetic dsRNA (poly(I:C)) leads to production of IFNβ [49]. It is likely that IFNβ influenced the expression of the genes mentioned above, particularly that supernatants from Bomac cells cultured in the presence of poly(I:C) contained a detectable amount of IFNβ measured at 8 h of stimulation (data not shown) compared to cells stimulated with CpG DNA. Sivori et al. [50] studied CpG DNA and poly(I:C) in regard to their ability to stimulate NK cells through TLRs and found that both could stimulate NK cells to acquire cytotoxic activity against tumor cells. However, poly(I:C) induced more robust responses than CpG DNA, because poly(I:C)-stimulated NK cells produced more IFNγ and TNFα and were highly cytotoxic compared to CpG DNA-stimulated NK cells. The fact that pro-inflammatory cytokines are released was shown in the study by Casey et al. [7] involving bovine MΦs infected with MAP where IL-1 was produced as early as 10 min under their experimental conditions.

The canonical pathways and networks, molecular and cellular functions as identified by IPA analysis of DE genes differed greatly between CpG DNA and poly(I:C) stimulated cells. In particular, CpG DNA promoted expression of genes involved in *free radical scavenging*, a group of molecules responsible for primary function of MΦs. Among the 21 genes in this group of molecules, 13 (*ABCA1, AGER, APLN, BNIP3, DDIT3, DDIT4, HK2, HSPB6, NR4A1, PLAU, PLAUR, S100A6, SREBF1*) were significantly upregulated with *p*-values ranging from 5.8 × 10^− 4^ to 1.45 × 10^− 8^. The downregulated genes in this function group could possibly be regarded as control mechanisms in the processes involving reactive oxygen species. In human MΦs *AGER* encodes a nucleic acid receptor that promotes inflammatory responses to DNA [51]. *BNIP3* and *DDIT4* are directly involved in generation of reactive oxygen species [52, 53] and formation of reactive oxygen species [54]. The remaining molecular and cellular functional groups in both stimulation groups mainly concerned cell development, morphology, survival, signalling or death. Further, on the molecular functions, careful examination of GO terms revealed that many top functions included chemokine activity. At least seven CXCL chemokines were found in both CpG DNA and poly(I:C) datasets. However, upregulation of *CXCL5, CXCL8, CXCL16* was observed only in poly(I:C)-stimulated Bomac cells and had an unaltered expression in CpG DNA-stimulated Bomac cells with the exception of *CXCL5*, which was also upregulated in CpG DNA-stimulated cells. *CXCL5* has been reported to activate MΦ and also increases the expression of *ABCA1* (mRNA upregulated in this dataset) [55].

Although we show marked differences in the transcriptomic landscape of MΦs treated with a viral or a bacterial PAMP, we cannot generalize the differences or compare them to in vivo conditions because, first, the CpG DNA and poly(I:C) used are highly purified reagents much different from live pathogens; second, we have used a transformed cell line that may not accurately represent the transcriptomic changes that occur in vivo (compare [12]). However, the results obtained in this study are largely in line with what is known about MΦ stimulation through TLR and further provide an insight into the early phase of transcriptomic alterations in MΦs during initial interaction with agonists through their pattern recognition receptors.

## Conclusion

Very early following stimulation of Bomac, poly(I:C), more than CpG DNA, triggered signals that exerted a transcriptional profile suited to an antiviral response, whereas CpG DNA influenced genes important for the phagocytic processes. Besides influencing the genes related to immune function, CpG DNA highly affected many genes in metabolic pathways and other non-immune biological processes. Although both, poly(I:C) and CpG DNA, are proposed vaccine adjuvants, poly(I:C) appears to be more potent in setting up the inflammatory landscape required to induce an efficient immune response.

## Additional files

Additional file 1: **Figure S1.** PlasmoTest results from Bomac cells. Supernatants were collected from 8 subsequent passages of Bomac cells and stored at − 80 °C. PlasmoTest (Invivogen, USA) was used to detect the presence of Mycoplasma, according to the kit manufacturer’s instructions. –C, negative control; +C, positive control; S, samples passages 1–8. Blue/purple color indicates positive signal, pink indicates negative signal. **Figure S2.** RT-PCR for the detection of BVDV in Bomac cells. The RT-PCR protocol used is that described by Katsuyoshi U. et al. J. Vet. Med.Sci. 60(7):867–870, 1998 with modification regarding enzymes used for reverse transcriptase and PCR. MW, molecular weight marker; 1–8, Bomac passages from 1 to 8; −, negative control; +, positive control. **Figure S3.** Upper panel: CD44 expression on the surface of BoMac cells. Red fluorescence – CD44; blue fluorescence – DNA. Scale bars = 20 μm. Lower panel: uptake of *Staphylococcus aureus* bioparticles conjugated with FITC by BoMac cells. As a positive control of bioparticles uptake, fresh bovine blood monocytes were used. Cells were incubated with *S. aureus* bioparticles for 1 h at 37 °C. Green fluorescence – bacteria, blue fluorescence – DNA. Arrows indicate phagocytosed bacteria. Scale bars = 20 μm. (DOCX 1300 kb)

Additional file 2: RNA integrity check before RNASeq analysis. (DOCX 87 kb)

Additional file 3: KEGG pathways generated for poly(I:C)vs Con, CpG DNA vs Con, and poly(I:C) vs CpG DNA (XLSX 98 kb)

Additional file 4: GO terms generated for poly(I:C)vs Con, CpG DNA vs Con, and poly(I:C) vs CpG DNA (XLSX 52 kb)

Additional file 5: *TNF* and *IRF7* Upstream regulated genes by CpG and poly(I:C), respectively (XLS 35 kb)

Additional file 6: **(A)** Top Network generated from the poly(I:C)vs CpG DNA comparison. Antimicrobial response, Inflammatory response, Cell-to-cell signalling and interaction, **(B)** Functional networks, **(C)** Upstream Regulators in poly(I:C) vs CpG DNA dataset. **(D)** Top Regulator Effect Network generated from the poly(I:C) vs CpG DNA. (DOCX 2230 kb)

Additional file 7: Tables. **(A)** Top 5 Canonical pathways generated by Ingenuity Pathway Analysis (IPA) of differentially expressed genes in Bomac cells stimulated with PAMPs poly(I:C) vs CpG dataset, **(B)** Top Networks generated in Bomac cell line treated from the comparison of poly(I:C) vs CpG DNA, **(C)** Top 5 Molecular and Cellular Functions identified in the differentially expressed genes from the poly(I:C) vs CpG DNA comparison, **(D)** Upstream Regulators identified in poly(I:C) vs CpG DNA comparison, **(E)** Top Regulator Effect Networks generated from poly(I:C) vs CpG DNA comparison. (DOCX 17 kb)

## Abbreviations

ABCA1: ATP binding cassette subfamily A member 1
ADAR: Adenosine deaminase, RNA specific
AGER: Advanced glycosylation end-product specific receptor
APLN: Apelin
APOBEC3B: Apolipoprotein B mRNA editing enzyme catalytic subunit 3B
BNIP3: BCL2 Interacting protein 3
CCL5: C-C Motif chemokine ligand 5
CD3G: CD3g molecule
CXCL16: C-X-C motif chemokine ligand 16
CXCL5: C-X-C motif chemokine ligand 5
CXCL8: C-X-C motif chemokine ligand 8
CYP1A1: Cytochrome P450 family 1 subfamily A member 1
DDIT3: DNA Damage inducible transcript 3
DDIT4: DNA Damage inducible transcript 4
DDX58: DExD/H-box helicase 58
EIF2AK2: Eukaryotic translation initiation factor 2 alpha kinase 2
FOXF1: Forkhead box F1
GITRL: Glucocorticoid-induced tnfr-related protein ligand (TNFSF18)
GNB3: G protein subunit beta 3
GSDMB: Gasdermin B
HDACG: Histone deacetylase gene
HIF1A: Hypoxia inducible factor 1 alpha subunit
HK2: Hexokinase 2
HLA-DMB: Major histocompatibility complex, class II, DM beta
HSPB6: Heat shock protein family B (small) member 6
IFI35: Interferon induced protein 35
IFI44L: Interferon induced protein 44 like
IFI6: Interferon alpha inducible protein 6
IFIT1: Interferon induced protein with tetratricopeptide repeats 1
IFIT3: Interferon induced protein with tetratricopeptide repeats 3
IFITM1: Interferon induced transmembrane protein 1
IFNA2: Interferon alpha 2A
IFNL1: Interferon lambda 1
IGFLR1: IGF like family receptor 1
IKKα: I kappa b kinase (conserved helix-loop-helix ubiquitous kinase)
IRAK1: interleukin 1 receptor associated kinase 1
IRAK4: interleukin 1 receptor associated kinase 4
IRF1: interferon regulatory factor 1
IRF7: interferon regulatory factor 7
IRF9: interferon regulatory factor 9
ISG15: interferon-stimulated protein 15 KDa, ubiquitin-like modifier
ISG20: interferon stimulated exonuclease gene 20
LBH: limb bud and heart development
MIST1R: macrophage stimulating 1 receptor
MX1: MX dynamin like GTPase 1
MX2: MX dynamin like GTPase 2
MyD88: myeloid differentiation primary response 88
MYOZ2: myozenin 2
NFκBIA: nuclear factor kappa B inhibitor alpha
NR4A1: nuclear receptor subfamily 4 group A member 1
OAS1: 2′-5′-oligoadenylate synthetase 1
OAS2: 2′-5′-oligoadenylate synthetase 2
OPN: opsin
PI3Kδ: phosphatidylinositol-4,5-bisphosphate 3-kinase catalytic subunit delta
PLAU: plasminogen activator, urokinase
PLUAR: plasminogen activator, urokinase receptor
PRKCB: protein kinase C beta
PRL: prolactin
PSMB8: proteasome subunit beta 8
RNASEL: ribonuclease L
RRAD: RRAD, Ras related glycolysis inhibitor and calcium channel regulator
RSAD2: radical S-adenosyl methionine domain containing 2
S100A6: S100 calcium binding protein A6
SLC26A10: solute carrier family 26 member 10
SOCS1: suppressor of cytokine signaling 1
SREBF1: sterol regulatory element binding transcription factor 1
STAT1: signal transducer and activator of transcription 1
STAT2: signal transducer and activator of transcription 2
TAP1: transporter 1, ATP binding cassette subfamily B member
TGFB1: transforming growth factor beta 1
TNFSF10: TNF superfamily member 18
TNFSF18: TNF superfamily member 18
TRAF3: TNF receptor associated factor 3
TRAF6: TNF receptor associated factor 6
UNC93B: unc-93 homolog B1
ZC3HAV1: zinc finger CCCH-type containing, antiviral 1

## References

[1] Elsik CG, Tellam RL, Worley KC, Gibbs RA, Muzny DM, Weinstock GM, Adelson DL, Eichler EE, Elnitski L, Guigo R (2009). The genome sequence of taurine cattle: a window to ruminant biology and evolution. Science.

[2] Lee DH, Ghiasi H. Roles of m1 and m2 macrophages in herpes simplex virus-1 infectivity. J Virol. 2017;91(15):e00578-17. 10.1128/JVI.00578-17 PMC5512262 28490589

[3] Nalpas NC, Park SDE, Magee DA, Taraktsoglou M, Browne JA, Conlon KM, Rue-Albrecht K, Killick KE, Hokamp K, Lohan AJ (2013). Whole-transcriptome, high-throughput rna sequence analysis of the bovine macrophage response to mycobacterium bovis infection in vitro. BMC Genomics.

[4] Magee DA, Taraktsoglou M, Killick KE, Nalpas NC, Browne JA, Park SDE, Conlon KM, Lynn DJ, Hokamp K, Gordon SV (2012). Global gene expression and systems biology analysis of bovine monocyte-derived macrophages in response to in vitro challenge with mycobacterium bovis. PLoS One.

[5] Lewandowska-Sabat AM, Boman GM, Downing A, Talbot R, Storset AK, Olsaker I (2013). The early phase transcriptome of bovine monocyte-derived macrophages infected with staphylococcus aureus in vitro. BMC Genomics.

[6] Lamont EA, Xu WW, Sreevatsan S (2013). Host-mycobacterium avium subsp. Paratuberculosis interactome reveals a novel iron assimilation mechanism linked to nitric oxide stress during early infection. BMC Genomics.

[7] Casey ME, Meade KG, Nalpas NC, Taraktsoglou M, Browne JA, Killick KE, Park SDE, Gormley E, Hokamp K, Magee DA (2015). Analysis of the bovine monocyte-derived macrophage response to mycobacterium avium subspecies paratuberculosis infection using rna-seq. Front Immunol.

[8] Kawasaki T, Kawai T (2014). Toll-like receptor signaling pathways. Front Immunol.

[9] Perales-Linares R, Navas-Martin S (2013). Toll-like receptor 3 in viral pathogenesis: friend or foe?. Immunology.

[10] Guiducci C, Coffman RL, Barrat FJ (2009). Signalling pathways leading to ifn-α production in human plasmacytoid dendritic cell and the possible use of agonists or antagonists of tlr7 and tlr9 in clinical indications. J Intern Med.

[11] Stabel JR, Stabel TJ (1995). Immortalization and characterization of bovine peritoneal macrophages transfected with sv40 plasmid DNA. Vet Immunol Immunopathol.

[12] Sager H, Davis WC, Jungi TW (1999). Bovine monocytoid cells transformed to proliferate cease to exhibit lineage-specific functions. Vet Immunol Immunopathol.

[13] Bürgi N, Josi C, Bürki S, Schweizer M, Pilo P (2018). Mycoplasma bovis co-infection with bovine viral diarrhea virus in bovine macrophages. Vet Res.

[14] Dolega P, Szulc-Dąbrowska L, Bossowska M, Mielcarska M, Nowak Z, Toka FN (2017). Innate immune gene transcript level associated with the infection of macrophages with ectromelia virus in two different mouse strains. Viral Immunol.

[15] Lawrence M, Gentleman R, Carey V (2009). Rtracklayer: an r package for interfacing with genome browsers. Bioinformatics.

[16] R: A language and environment for statistical computing. https://www.R-project.org. Accessed December 2017.

[17] Dobin A, Davis CA, Schlesinger F, Drenkow J, Zaleski C, Jha S, Batut P, Chaisson M, Gingeras TR (2013). Star: Ultrafast universal rna-seq aligner. Bioinformatics.

[18] Liao Y, Smyth GK, Shi W (2014). Featurecounts: an efficient general purpose program for assigning sequence reads to genomic features. Bioinformatics.

[19] Liao Y, Smyth GK, Shi W (2013). The subread aligner: fast, accurate and scalable read mapping by seed-and-vote. Nucleic Acids Res.

[20] Huber W, Carey VJ, Gentleman R, Anders S, Carlson M, Carvalho BS, Bravo HC, Davis S, Gatto L, Girke T (2015). Orchestrating high-throughput genomic analysis with bioconductor. Nat Methods.

[21] Robinson MD, McCarthy DJ, Smyth GK (2010). Edger: a bioconductor package for differential expression analysis of digital gene expression data. Bioinformatics.

[22] Chen Y, Lun A, Smyth G (2016). From reads to genes to pathways: differential expression analysis of rna-seq experiments using rsubread and the edger quasi-likelihood pipeline [version 2; referees: 5 approved]. F1000Res.

[23] Lun AT, Chen Y, Smyth GK (2016). It’s de-licious: a recipe for differential expression analyses of rna-seq experiments using quasi-likelihood methods in edger. Methods Mol Biol.

[24] Robinson MD, Oshlack A (2010). A scaling normalization method for differential expression analysis of rna-seq data. Genome Biol.

[25] Benjamini Y, Hochberg Y (1995). Controlling the false discovery rate: a practical and powerful approach to multiple testing. J R Stat Soc Series B Stat Methodol.

[26] Young MD, Wakefield MJ, Smyth GK, Oshlack A (2010). Gene ontology analysis for rna-seq: accounting for selection bias. Genome Biol.

[27] Luo W, Brouwer C (2013). Pathview: an r/bioconductor package for pathway-based data integration and visualization. Bioinformatics.

[28] Krämer A, Green J, Pollard J, Tugendreich S (2014). Causal analysis approaches in ingenuity pathway analysis. Bioinformatics.

[29] Chase CC, Thakur N, Darweesh MF, Morarie-Kane SE, Rajput MK (2015). Immune response to bovine viral diarrhea virus--looking at newly defined targets. Anim Health Res Rev.

[30] Joetham A, Ohnishi H, Okamoto M, Takeda K, Schedel M, Domenico J, Dakhama A, Gelfand EW (2012). Loss of t regulatory cell suppression following signaling through glucocorticoid-induced tumor necrosis receptor (gitr) is dependent on c-Jun n-terminal kinase activation. J Biol Chem.

[31] Hanabuchi S, Watanabe N, Wang Y-H, Wang Y-H, Ito T, Shaw J, Cao W, Qin FX-F, Liu Y-J (2006). Human plasmacytoid predendritic cells activate nk cells through glucocorticoid-induced tumor necrosis factor receptor-ligand (gitrl). Blood.

[32] Gao J, Wang D, Liu D, Liu M, Ge Y, Jiang M, Liu Y, Zheng D (2015). Tumor necrosis factor–related apoptosis-inducing ligand induces the expression of proinflammatory cytokines in macrophages and re-educates tumor-associated macrophages to an antitumor phenotype. Mol Biol Cell.

[33] Oka N, Nakahara S, Takenaka Y, Fukumori T, Hogan V, H-o K, Yanagawa T, Raz A (2005). Galectin-3 inhibits tumor necrosis factor–related apoptosis-inducing ligand–induced apoptosis by activating akt in human bladder carcinoma cells. Cancer Res.

[34] Androutsopoulos VP, Tsatsakis AM, Spandidos DA (2009). Cytochrome p450 cyp1a1: wider roles in cancer progression and prevention. BMC Cancer.

[35] Xu L, Zhou X, Wang W, Wang Y, Yin Y, LJWvd L, Sprengers D, Metselaar HJ, Peppelenbosch MP, Pan Q (2016). Ifn regulatory factor 1 restricts hepatitis e virus replication by activating stat1 to induce antiviral ifn-stimulated genes. FASEB J.

[36] Rabbani MAG, Ribaudo M, Guo J-T, Barik S (2016). Identification of interferon-stimulated gene proteins that inhibit human parainfluenza virus type 3. J Virol.

[37] Ranaware PB, Mishra A, Vijayakumar P, Gandhale PN, Kumar H, Kulkarni DD, Raut AA (2016). Genome wide host gene expression analysis in chicken lungs infected with avian influenza viruses. PLoS One.

[38] Lin R-J, Yu H-P, Chang B-L, Tang W-C, Liao C-L, Lin Y-L (2009). Distinct antiviral roles for human 2′,5′-oligoadenylate synthetase family members against dengue virus infection. J Immunol.

[39] Liu S-Y, Sanchez DJ, Aliyari R, Lu S, Cheng G (2012). Systematic identification of type i and type ii interferon-induced antiviral factors. Proc Natl Acad Sci U S A.

[40] Triantafyllopoulou A, Franzke C-W, Seshan SV, Perino G, Kalliolias GD, Ramanujam M, van Rooijen N, Davidson A, Ivashkiv LB (2010). Proliferative lesions and metalloproteinase activity in murine lupus nephritis mediated by type i interferons and macrophages. Proc Natl Acad Sci U S A.

[41] Pomeranz LE, Ekstrand MI, Latcha KN, Smith GA, Enquist LW, Friedman JM (2017). Gene expression profiling with cre-conditional pseudorabies virus reveals a subset of midbrain neurons that participate in reward circuitry. J Neurosci.

[42] Chao KL, Kulakova L, Herzberg O (2017). Gene polymorphism linked to increased asthma and ibd risk alters gasdermin-b structure, a sulfatide and phosphoinositide binding protein. Proc Natl Acad Sci U S A.

[43] Hu Y, Jin S, Cheng L, Liu G, Jiang Q (2017). Autoimmune disease variants regulate gsdmb gene expression in human immune cells and whole blood. Proc Natl Acad Sci U S A.

[44] Ekwall AKH, Whitaker JW, Hammaker D, Bugbee WD, Wang W, Firestein GS (2015). The rheumatoid arthritis risk gene lbh regulates growth in fibroblast-like synoviocytes. Arthritis rheumatol.

[45] Hanna RN, Shaked I, Hubbeling HG, Punt JA, Wu R, Herrley E, Zaugg C, Pei H, Geissmann F, Ley K (2012). Nr4a1 (nur77) deletion polarizes macrophages toward an inflammatory phenotype and increases atherosclerosis. Circulation Res.

[46] Ding N, Hah N, Yu RT, Sherman MH, Benner C, Leblanc M, He M, Liddle C, Downes M, Evans RM (2015). Brd4 is a novel therapeutic target for liver fibrosis. Proc Natl Acad Sci U S A.

[47] Flores-Espinosa P, Preciado-Martínez E, Mejía-Salvador A, Sedano-González G, Bermejo-Martínez L, Parra-Covarruvias A, Estrada-Gutiérrez G, Vega-Sánchez R, Méndez I, Quesada-Reyna B (2017). Selective immuno-modulatory effect of prolactin upon pro-inflammatory response in human fetal membranes. J Reprod Immunol.

[48] Medina-Estrada I, Alva-Murillo N, López-Meza JE, Ochoa-Zarzosa A (2015). Non-classical effects of prolactin on the innate immune response of bovine mammary epithelial cells: implications during staphylococcus aureus internalization. Microb Pathog.

[49] Uematsu S, Akira S (2007). Toll-like receptors and type i interferons. J Biol Chem.

[50] Sivori S, Falco M, Chiesa MD, Carlomagno S, Vitale M, Moretta L, Moretta A (2004). Cpg and double-stranded rna trigger human nk cells by toll-like receptors: induction of cytokine release and cytotoxicity against tumors and dendritic cells. Proc Natl Acad Sci U S A.

[51] Sirois CM, Jin T, Miller AL, Bertheloot D, Nakamura H, Horvath GL, Mian A, Jiang J, Schrum J, Bossaller L (2013). Rage is a nucleic acid receptor that promotes inflammatory responses to DNA. J Exp Med.

[52] Ghavami S, Eshragi M, Ande SR, Chazin WJ, Klonisch T, Halayko AJ, McNeill K, Hashemi M, Kerkhoff C, Los M (2010). S100a8/a9 induces autophagy and apoptosis via ros-mediated cross-talk between mitochondria and lysosomes that involves bnip3. Cell Res.

[53] Shoshani T, Faerman A, Mett I, Zelin E, Tenne T, Gorodin S, Moshel Y, Elbaz S, Budanov A, Chajut A (2002). Identification of a novel hypoxia-inducible factor 1-responsive gene, rtp801, involved in apoptosis. Mol Cell Biol.

[54] Vande Velde C, Cizeau J, Dubik D, Alimonti J, Brown T, Israels S, Hakem R, Greenberg AH (2000). Bnip3 and genetic control of necrosis-like cell death through the mitochondrial permeability transition pore. Mol Cell Biol.

[55] Rousselle A, Qadri F, Leukel L, Yilmaz R, Fontaine J-F, Sihn G, Bader M, Ahluwalia A, Duchene J (2013). Cxcl5 limits macrophage foam cell formation in atherosclerosis. J Clin Invest.

