# Supplementary material for: Bacterial and viral pathogen-associated molecular patterns induce divergent early transcriptomic landscapes in a bovine macrophage cell line
Source: BMC Genomics. 2019 Jan 8;20:15. doi: 10.1186/s12864-018-5411-5 (PMC6323673; doi:10.1186/s12864-018-5411-5)
Supplement: Supplementary file 1 — Figure S1. PlasmoTest results from Bomac cells. Supernatants were collected from 8 subsequent passages of Bomac cells and stored at − 80 °C. PlasmoTest (Invivogen, USA) was used to detect the presence of Mycoplasma, according to the kit manufacturer’s instructions. –C, negative control; +C, positive control; S, samples passages 1–8. Blue/purple color indicates positive signal, pink indicates negative signal. Figure S2. RT-PCR for the detection of BVDV in Bomac cells. The RT-PCR protocol used is that described by Katsuyoshi U. et al. J. Vet. Med.Sci. 60(7):867–870, 1998 with modification regarding enzymes used for reverse transcriptase and PCR. MW, molecular weight marker; 1–8, Bomac passages from 1 to 8; −, negative control; +, positive control. Figure S3. Upper panel: CD44 expression on the surface of BoMac cells. Red fluorescence – CD44; blue fluorescence – DNA. Scale bars = 20 μm. Lower panel: uptake of Staphylococcus aureus bioparticles conjugated with FITC by BoMac cells. As a positive control of bioparticles uptake, fresh bovine blood monocytes were used. Cells were incubated with S. aureus bioparticles for 1 h at 37 °C. Green fluorescence – bacteria, blue fluorescence – DNA. Arrows indicate phagocytosed bacteria. Scale bars = 20 μm. (DOCX 1300 kb) [file 12864_2018_5411_MOESM1_ESM.docx]

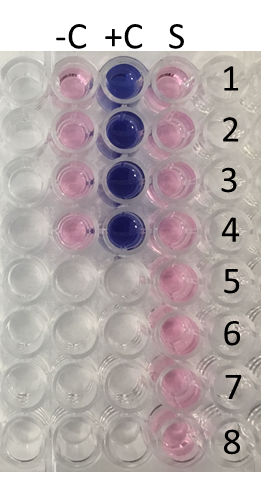


**Figure S1.** PlasmoTest results from Bomac cells. Supernatants were collected from 8 subsequent passages of Bomac cells and stored at -80°C. PlasmoTest (Invivogen, USA) was used to detect the presence of Mycoplasma, according to the kit manufacturer’s instructions. –C, negative control; +C, positive control; S, samples passages 1 -8. Blue/purple color indicates positive signal, pink indicates negative signal.


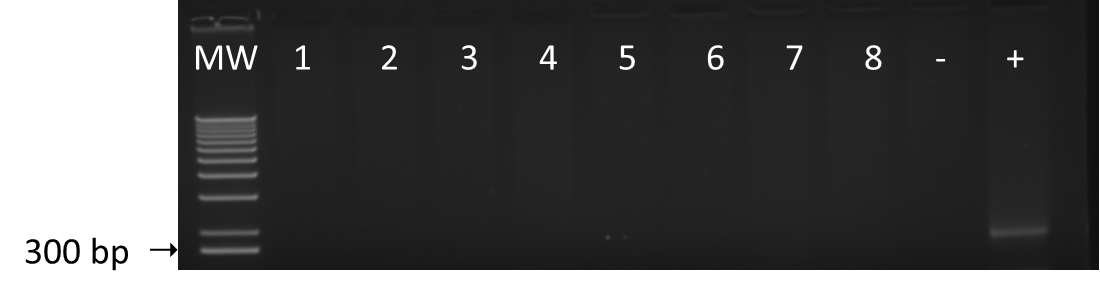


**Figure S2.** RT-PCR for the detection of BVDV in Bomac cells. The RT-PCR protocol used is that described by Katsuyoshi U. et al. J. Vet. Med.Sci. 60(7):867-870, 1998 with modification regarding enzymes used for reverse transcriptase and PCR. MW, molecular weight marker; 1 – 8, Bomac passages from 1-8; -, negative control; +, positive control.


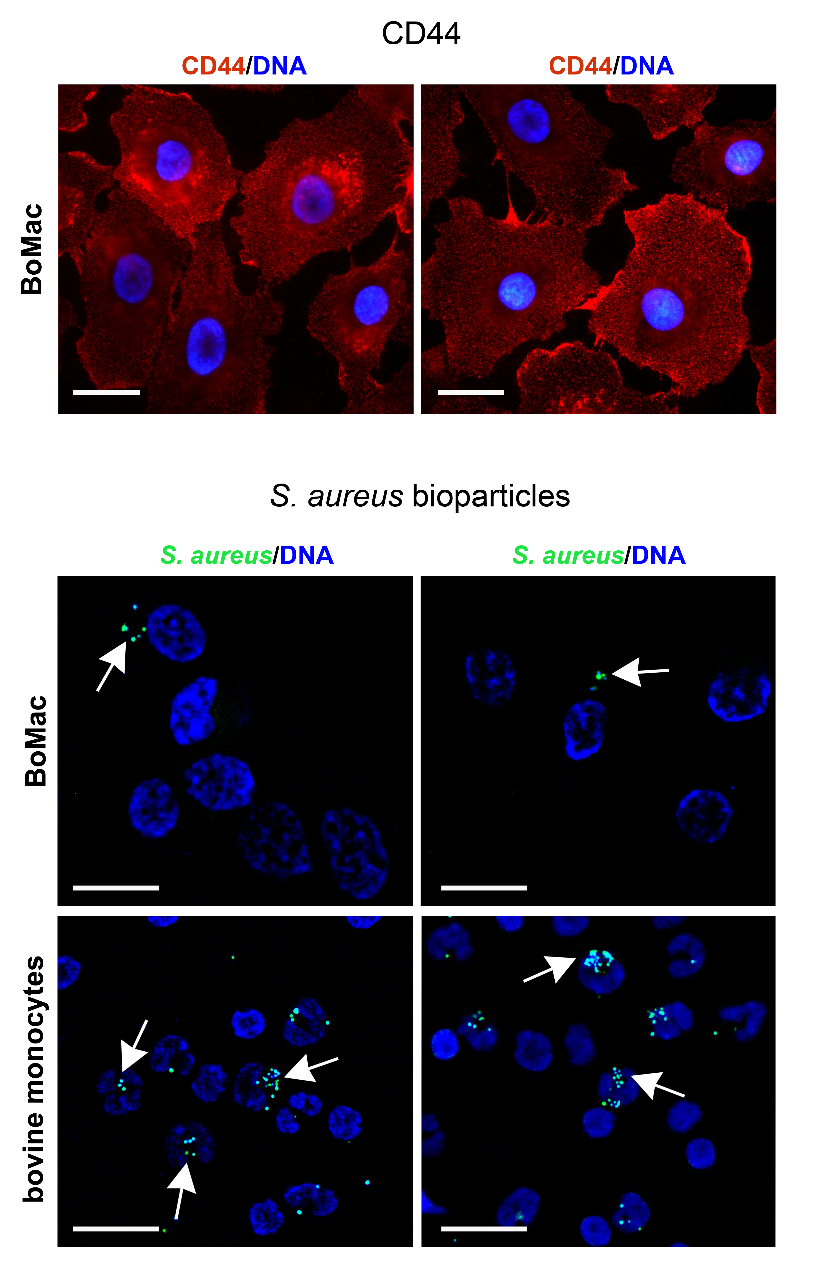


**Figure S3.** Upper panel: CD44 expression on the surface of BoMac cells. Red fluorescence – CD44; blue fluorescence – DNA. Scale bars = 20 µm.

Lower panel: uptake of *Staphylococcus aureus* bioparticles conjugated with FITC by BoMac cells. As a positive control of bioparticles uptake, fresh bovine blood monocytes were used. Cells were incubated with *S.aureus* bioparticles for 1 h at 37 °C. Green fluorescence – bacteria, blue fluorescence – DNA. Arrows indicate phagocytosed bacteria. Scale bars = 20 µm.
