# Supplementary material for: Bacterial and viral pathogen-associated molecular patterns induce divergent early transcriptomic landscapes in a bovine macrophage cell line
Source: BMC Genomics. 2019 Jan 8;20:15. doi: 10.1186/s12864-018-5411-5 (PMC6323673; doi:10.1186/s12864-018-5411-5)
Supplement: Supplementary file 2 — RNA integrity check before RNASeq analysis. (DOCX 87 kb) [file 12864_2018_5411_MOESM2_ESM.docx]

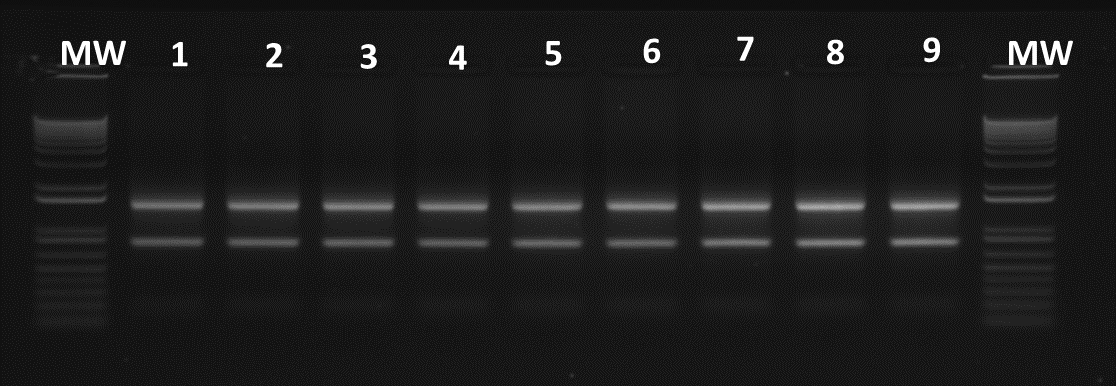


**Additional File 2**. RNA integrity monitoring. RNA was isolated from BoMac cells with Qiagen miRNeasy Isolation Kit. RNA was prepared by diluting 200 ng RNA in ultrapure water RNAse free and heated at 70°C for 1 min. and then mixed with Orange loading dye. Electrophoresis was run in 1xTAE buffer, at 50V for 45 minutes on 1.2% agarose gel containing ethidium bromide at 10 μg/ml. Results were visualized in Syngene Imaging apparatus. **MW**, Molecular weight marker; **Lane 1, 2**, **3**, Control BoMac cells; **Lane 4, 5, 6** BoMac cells stimulated with polyI:C at 10 ug/ml; **Lane 7, 8, 9** BoMac cells stimulated with CpG DNA at 50 ug/ml
