# Supplementary material for: Bacterial and viral pathogen-associated molecular patterns induce divergent early transcriptomic landscapes in a bovine macrophage cell line
Source: BMC Genomics. 2019 Jan 8;20:15. doi: 10.1186/s12864-018-5411-5 (PMC6323673; doi:10.1186/s12864-018-5411-5)

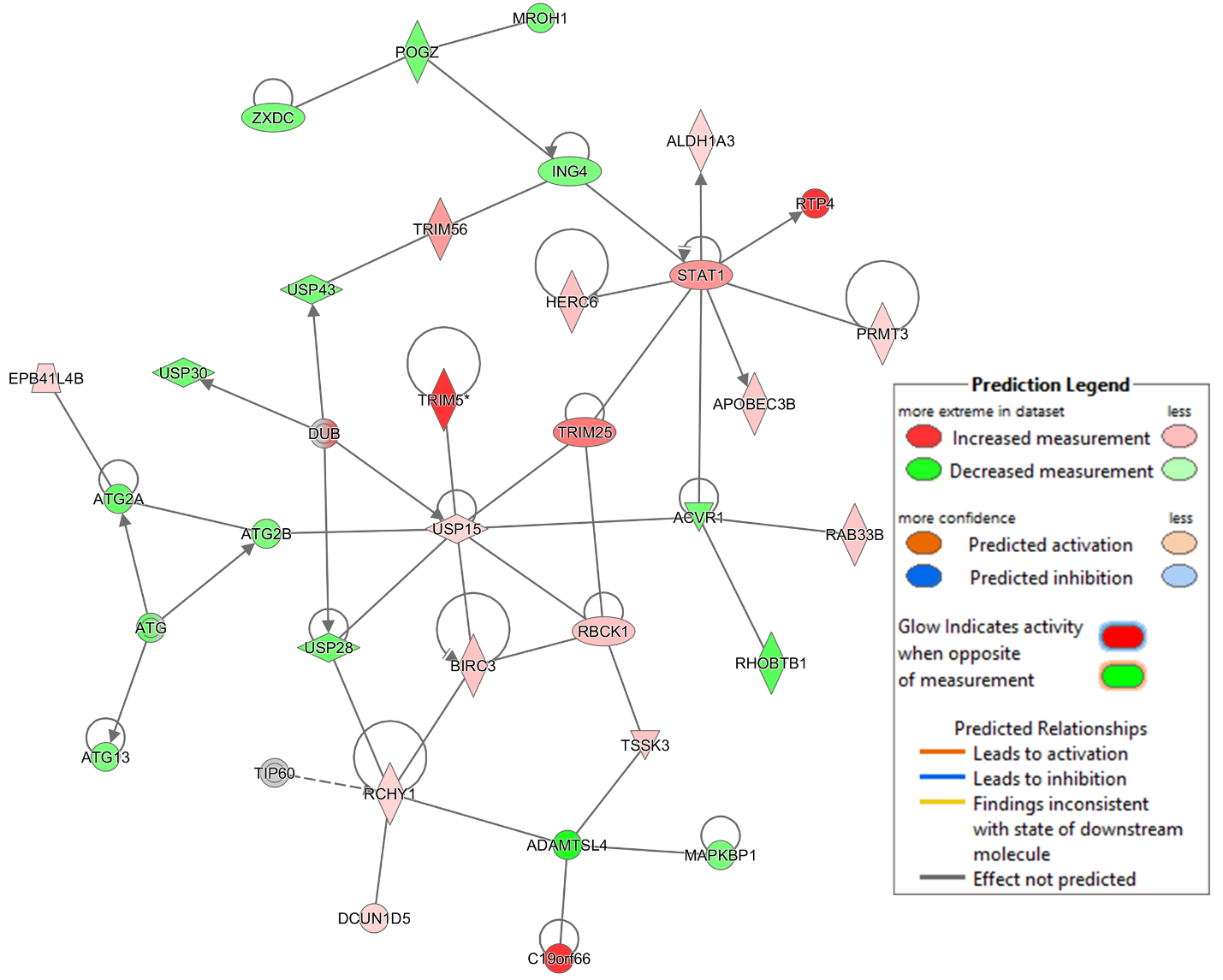
**Additional File 6.**

**(A)** Top Network generated from the pI:Cvs CpG DNA comparison. Antimicrobial response, Inflammatory response, Cell-to-cell signalling and interaction,

**(B)** Functional networks. Apoptosis of macrophages.


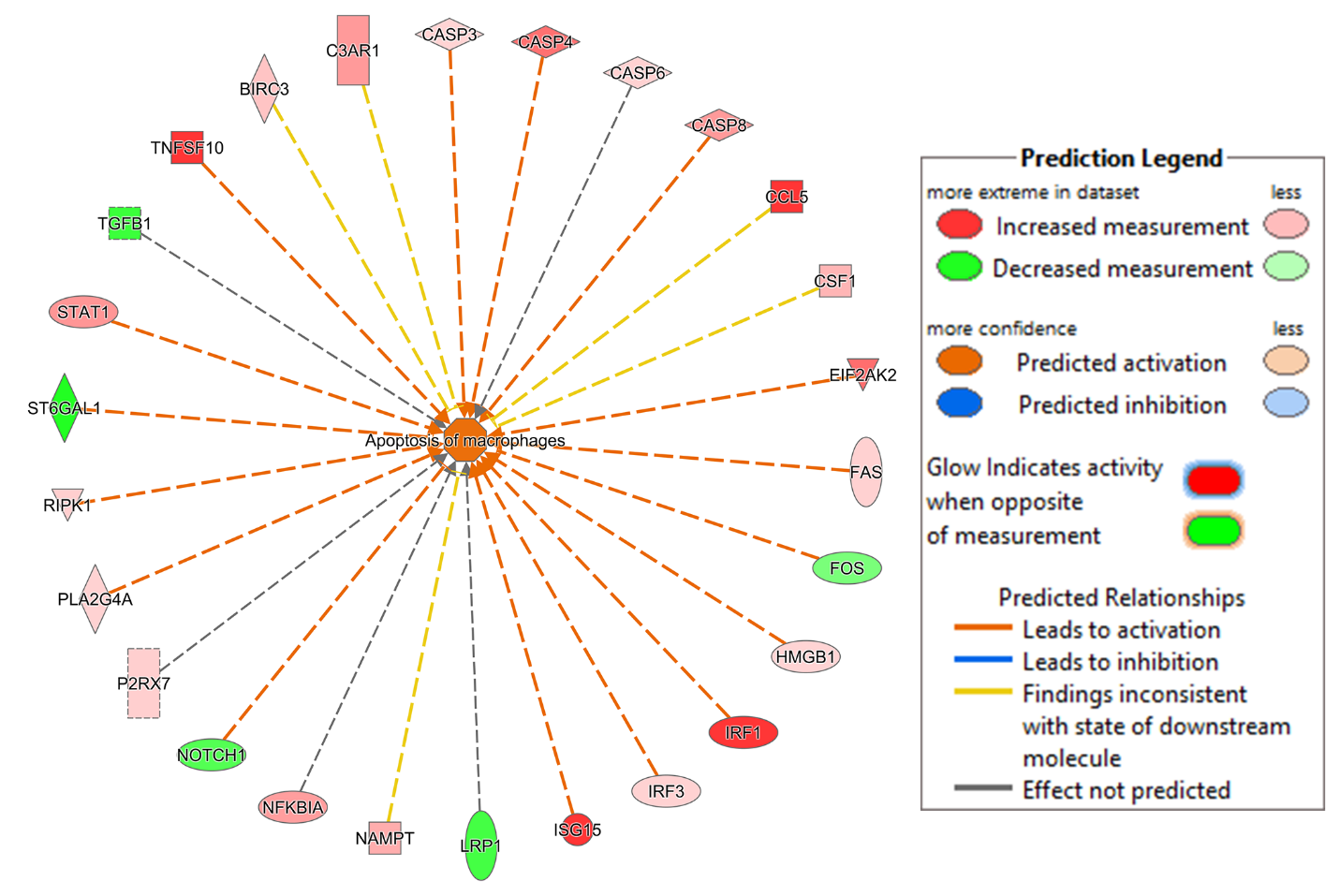


**(C)** Upstream Regulators in pI:Cvs CpG DNA dataset. Interferon.


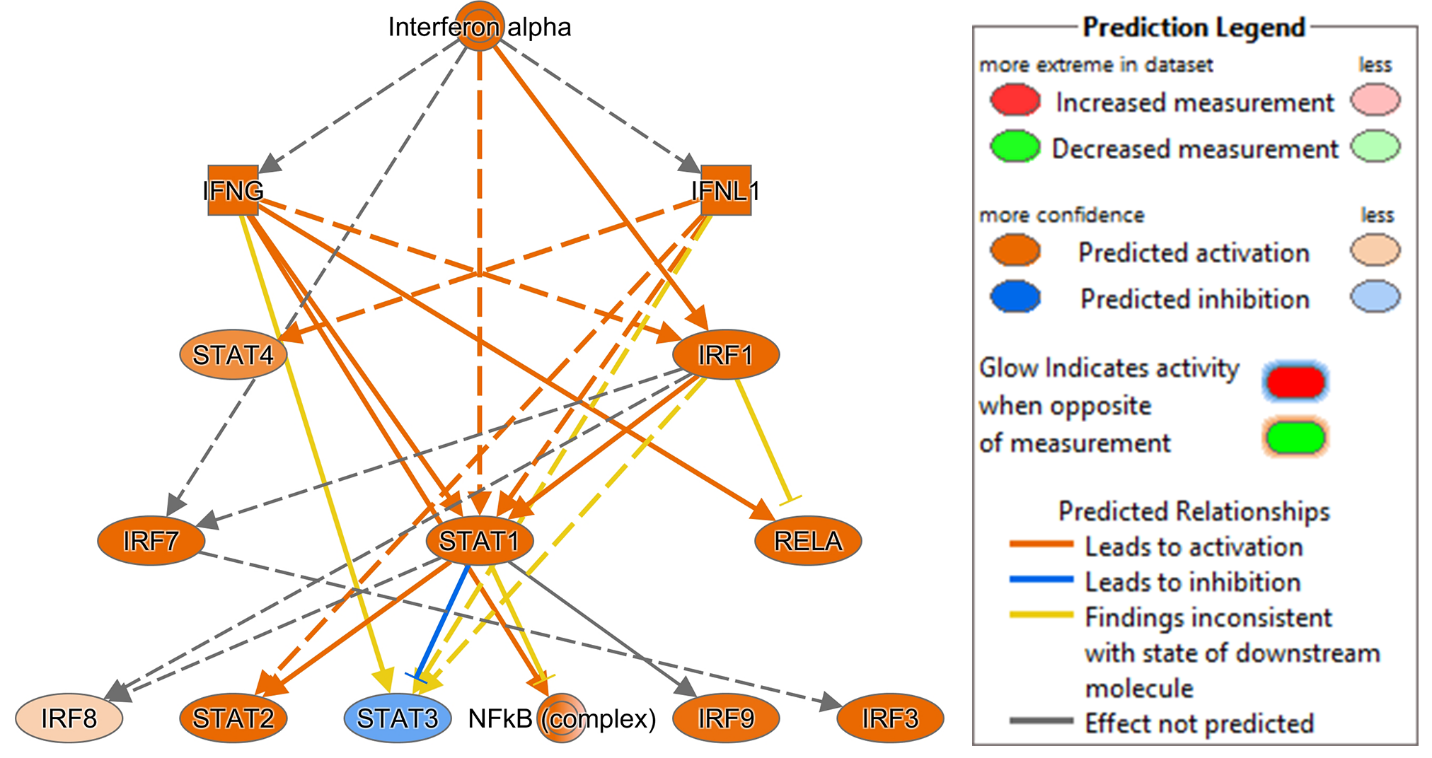


**(D)** Top Regulator Effect Network generated from the pI:Cvs CpG DNA. Prolactin.


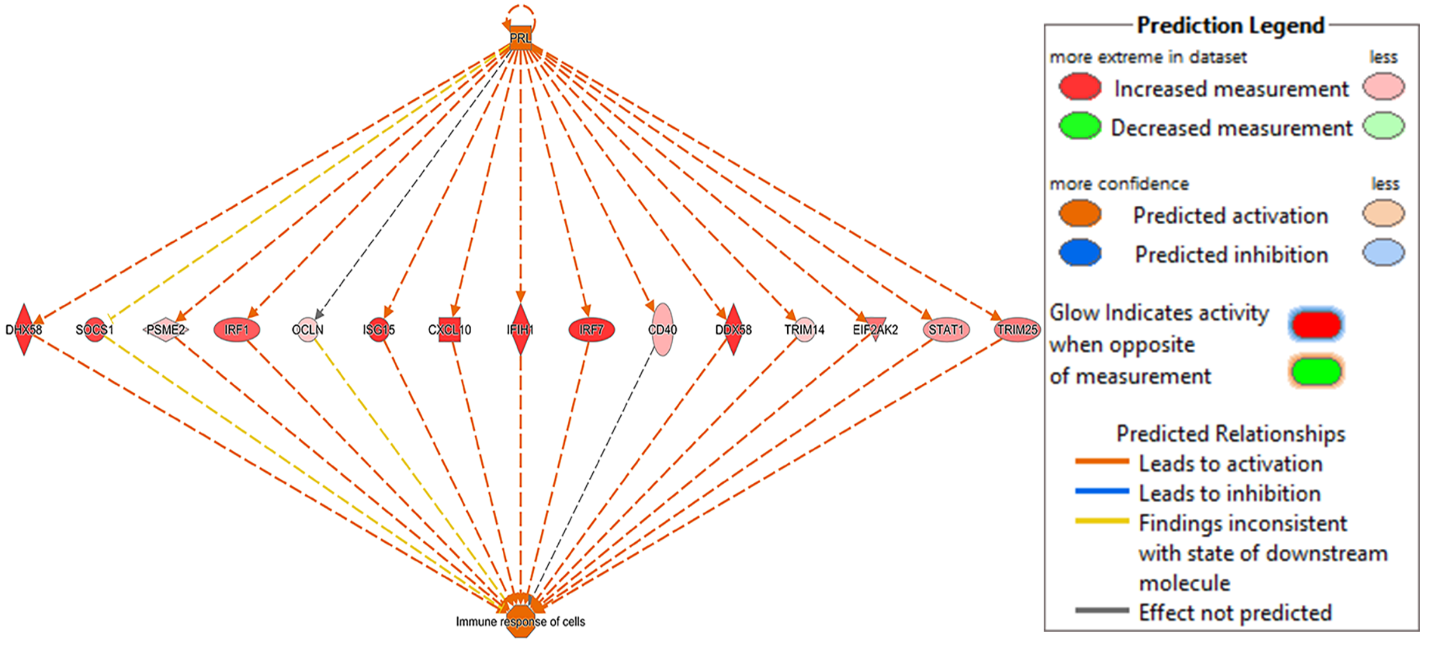

Supplement: Supplementary file 6 — (A) Top Network generated from the poly(I:C)vs CpG DNA comparison. Antimicrobial response, Inflammatory response, Cell-to-cell signalling and interaction, (B) Functional networks, (C) Upstream Regulators in poly(I:C) vs CpG DNA dataset. (D) Top Regulator Effect Network generated from the poly(I:C) vs CpG DNA. (DOCX 2230 kb) [file 12864_2018_5411_MOESM6_ESM.docx]
