# Supplementary material for: Bacterial and viral pathogen-associated molecular patterns induce divergent early transcriptomic landscapes in a bovine macrophage cell line
Source: BMC Genomics. 2019 Jan 8;20:15. doi: 10.1186/s12864-018-5411-5 (PMC6323673; doi:10.1186/s12864-018-5411-5)
Supplement: Supplementary file 7 — Tables. (A) Top 5 Canonical pathways generated by Ingenuity Pathway Analysis (IPA) of differentially expressed genes in Bomac cells stimulated with PAMPs poly(I:C) vs CpG dataset, (B) Top Networks generated in Bomac cell line treated from the comparison of poly(I:C) vs CpG DNA, (C) Top 5 Molecular and Cellular Functions identified in the differentially expressed genes from the poly(I:C) vs CpG DNA comparison, (D) Upstream Regulators identified in poly(I:C) vs CpG DNA comparison, (E) Top Regulator Effect Networks generated from poly(I:C) vs CpG DNA comparison. (DOCX 17 kb) [file 12864_2018_5411_MOESM7_ESM.docx]

1. Top 5 Canonical pathways generated by Ingenuity Pathway Analysis (IPA) of differentially expressed genes in BoMac cells stimulated with PAMPs pI:C vs CpG dataset.

| **Canonical Pathway** | ***p*-Value** | **Ratio** | **Molecules** |
| --- | --- | --- | --- |
| Interferon Signaling | 4.39x10^-12^ | 0.472 | SOCS1, IFIT3, OAS1, PTPN2, MX1, IFI35, IRF9, PSMB8, IFNAR2, TAP1, IRF1, ISG15, IFIT1, STAT2, IFI6, STAT1, IFITM1 |
| Death Receptor Signaling | 1.30x10^-10^ | 0.269 | MAP3K14, CASP3, NFKBIE, PARP10, ZC3HAV1, TNFSF10, PARP12, TBK1, NFKB2, NFKB1, PARP9, FAS, TANK, DAXX, CASP6, NFKBIA, RIPK1, ACTA2, HTRA2, HSPB7, ACTG2, CASP8, BIRC3, CASP7, PARP14 |
| Activation of IRF by Cytosolic Pattern Recognition Receptors | 3.55x10^-10^ | 0.317 | DHX58, NFKBIE, ZBP1, IRF9, TBK1, NFKB2 ,IRF3, ADAR, NFKB1, ISG15, IFIH1, TANK, IRF7, RIPK1, NFKBIA, CD40, DDX58, STAT2, STAT1 ,IFIT2 |
| Hepatic Fibrosis / Hepatic Stellate Cell Activation | 4.19x10^-10^ | 0.193 | COL8A2, MYH10 ,MYH6, ICAM1, CTGF, COL4A6, FGF2, COL4A3, VEGFB, COL8A1, COL4A2, CCL5, NFKB1, FAS, COL15A1, COL16A1, COL1A2, EDN1, TGFB1, TGFB2, SERPINE1, STAT1, COL27A1, CXCL8, COL12A1, SMAD7, MMP2, NFKB2, IFNAR2, COL1A1, IGF2, CD40, ACTA2, CSF1, TGFB3, COL11A1 |
| TWEAK Signaling | 5.22E-08 | 0.371 | MAP3K14 ,CASP3, NFKBIE, NFKB2 ,NFKB1, TNFRSF12A, CASP6, NFKBIA ,RIPK1, CASP8, BIRC3, CASP7, TRAF1 |

1. Top Networks generated in BoMac cell line treated from the comparison of pI:C vs CpG DNA.

| **Associated Network Functions** | **Score** |
| --- | --- |
| Cancer, Gastrointestinal Disease, Organismal Injury and Abnormalities | 40 |
| Antimicrobial Response, Inflammatory Response, Cell-To-Cell Signaling and Interaction | 40 |
| Molecular Transport, Energy Production, Nucleic Acid Metabolism | 40 |
| Cell Death and Survival, Cellular Function and Maintenance, Hereditary Disorder | 37 |
| Developmental Disorder, Hereditary Disorder, Metabolic Disease | 37 |

1. Top 5 Molecular and Cellular Functions identified in the differentially expressed genes from the pI:C vs CpG DNA comparison.

| **Name** | ***p*-value** | **#Molecules** |
| --- | --- | --- |
| Cell Death and Survival | 3.68x10^-5^ - 2.62x10^-22^ | 469 |
| Cellular Movement | 3.08x10^-5^ - 1.34x10^-19^ | 334 |
| Gene Expression | 1.14x10^-5^ - 1.44x10^-16^ | 342 |
| Cell Cycle | 1.14x10^-5^ - 8.63x10^-12^ | 193 |
| Cellular Function and Maintenance | 3.40x10^-5^ - 1.06x10^-11^ | 405 |

1. Upstream Regulators identified in pI:C vs CpG DNA comparison.

| **Upstream Regulator** | ***p*-value of overlap** |
| --- | --- |
| Interferon alpha | 2.01x10^-34^ |
| IRF7 | 1.62x10^-30^ |
| IFNG | 3.69x10^-30^ |
| poly rI:rC-RNA | 3.70x10^-30^ |
| IFNL1 | 1.06x10^-27^ |

1. Top Regulator Effect Networks generated from pI:C vs CpG DNA comparison.

| **Regulators** | **Diseases & Functions** | **Consistency Score** |
| --- | --- | --- |
| ribavirin | Viral Infection | 3.615 |
| 2 lipopolysaccharide | Replication of viral replicon | 3.606 |
| 3 PRL | Immune response of cells | 3.5 |
| 4 lipopolysaccharide | Viral life cycle | 3.474 |
| 5 IFNA2 | Replication of viral replicon | 3.464 |
